# Digitalized Cognitive Behavioral Interventions for Depressive Symptoms During Pregnancy: Systematic Review

**DOI:** 10.2196/33337

**Published:** 2022-02-23

**Authors:** Wan Mohd Azam Wan Mohd Yunus, Hanna-Maria Matinolli, Otto Waris, Subina Upadhyaya, Miika Vuori, Tarja Korpilahti-Leino, Terja Ristkari, Tarja Koffert, Andre Sourander

**Affiliations:** 1 Research Centre for Child Psychiatry University of Turku Turku Finland; 2 INVEST Research Flagship University of Turku Turku Finland; 3 Department of Psychology, School of Human Resource Development & Psychology Faculty of Social Sciences & Humanities Universiti Teknologi Malaysia Johor Malaysia; 4 Turku University of Applied Sciences Turku Finland; 5 Department of Teacher Education Turku Institute for Advanced Studies University of Turku Turku Finland; 6 Turku University Hospital Turku Finland

**Keywords:** pregnancy, antenatal depression, systematic review, cognitive behavior therapy, digital interventions, COVID-19

## Abstract

**Background:**

Studies have shown a high prevalence of depression during pregnancy, and there is also evidence that cognitive behavioral therapy (CBT) is one of the most effective psychosocial interventions. Emerging evidence from randomized controlled trials (RCTs) has shown that technology has been successfully harnessed to provide CBT interventions for other populations. However, very few studies have focused on their use during pregnancy. This approach has become increasingly important in many clinical areas due to the COVID-19 pandemic, and our study aimed to expand the knowledge in this particular clinical area.

**Objective:**

Our systematic review aimed to bring together the available research-based evidence on digitalized CBT interventions for depression symptoms during pregnancy.

**Methods:**

A systematic review of the Web of Science, Cochrane Central Register of Controlled Trials, CINAHL, MEDLINE, Embase, PsycINFO, Scopus, ClinicalTrials.gov, and EBSCO Open Dissertations databases was carried out from the earliest available evidence to October 27, 2021. Only RCT studies published in English were considered. The PRISMA (Preferred Reporting Items of Systematic Reviews and Meta-analyses) guidelines were followed, and the protocol was registered on the Prospective Register of Systematic Reviews. The risk of bias was assessed using the revised Cochrane risk-of-bias tool for randomized trials.

**Results:**

The review identified 7 studies from 5 countries (the United States, China, Australia, Norway, and Sweden) published from 2015 to 2021. The sample sizes ranged from 25 to 1342 participants. The interventions used various technological elements, including text, images, videos, games, interactive features, and peer group discussions. They comprised 2 guided and 5 unguided approaches. Using digitalized CBT interventions for depression during pregnancy showed promising efficacy, with guided intervention showing higher effect sizes (Hedges g=1.21) than the unguided interventions (Hedges g=0.14-0.99). The acceptability of the digitalized CBT interventions was highly encouraging, based on user feedback. Attrition rates were low for the guided intervention (4.5%) but high for the unguided interventions (22.1%-46.5%). A high overall risk of bias was present for 6 of the 7 studies.

**Conclusions:**

Our search only identified a small number of digitalized CBT interventions for pregnant women, despite the potential of this approach. These showed promising evidence when it came to efficacy and positive outcomes for depression symptoms, and user feedback was positive. However, the overall risk of bias suggests that the efficacy of the interventions needs to be interpreted with caution. Future studies need to consider how to mitigate these sources of biases. Digitalized CBT interventions can provide prompt, effective, evidence-based interventions for pregnant women. This review increases our understanding of the importance of digitalized interventions during pregnancy, including during the COVID-19 pandemic.

**Trial Registration:**

PROSPERO International Prospective Register of Systematic Reviews CRD42020216159; https://www.crd.york.ac.uk/prospero/display_record.php?RecordID=216159

## Introduction

It is estimated that one-fifth of mothers experience depression during pregnancy [1]. This has been strongly associated with postnatal depression [2,3] and linked with several adverse health and psychosocial outcomes for both the mother and child. These include preterm delivery [4,5], difficulties in mother-child interactions, impaired cognitive and psychomotor development [6], and altered fetal brain development [7,8]. Pregnant women experience changes to psychological symptoms due to complex biological and psychological interactions throughout the pregnancy period [9]. Therefore, recognizing depression symptoms during pregnancy at an early stage and preventing future risk of depression should be considered an important global public health challenge.

There is evidence that psychosocial treatment, cognitive behavioral therapy (CBT), and interpersonal psychotherapy are effective in treating depression during pregnancy [10]. The US Preventive Services Task Force [11] reviewed pharmacological and nonpharmacological interventions for depression during pregnancy and postnatal periods. It reported that CBT was effective and stated that global studies had not reported harmful outcomes for either mothers or infants. A randomized controlled trial (RCT) of 217 pregnant women in the United States showed that CBT significantly reduced depression during pregnancy when it was compared with standard care [12]. Concerns about the safety of using selective serotonin reuptake inhibitors during pregnancy and their effect on offspring have been reported, such as altering brain circuits [13], increasing the risk for later depression [14], and increased problems with motor and language development [15]. These highlight the need to study psychosocial interventions.

There is huge gap between what pregnant women need and the human resources that are available to provide such services. Very few women seek help with mental health issues during pregnancy [16]. Barriers to seeking help have included individual factors, such as lack of motivation, time constraints, or they just decided not to seek care [16,17]. Social factors have included stigma and lack of social support, and practical issues have included lack of money or childcare. There are also structural barriers, like lack of information about services, treatment provider responsivity, and treatment accessibility issues [16-20]. The World Health Organization’s Mental Health Atlas also reported unequal and limited availability of mental health services and resources across the world [21]. Depression is common during pregnancy, and it needs to be treated without delay. The Institute of Medicine recognized the different spectrum of interventions for mental health based on the risk for the target population: prevention (universal, selective, indicated), treatment (case identification, standard treatment for known disorders), and maintenance (compliance with long-term treatment, aftercare) [22]. Focusing on pregnant women with different depression risk levels is essential, given that less than three-quarters of pregnant women are screened for depression [23].

Digitalized interventions provide an effective way of reaching people and have the potential to overcome barriers, such as the fear of being stigmatized, practical reasons, and treatment availability [24-28]. Digitalized interventions are also becoming increasingly important, as mental health services struggle to deal with the considerable increase in demand for services due to the COVID-19 pandemic. This health emergency began at a time when resources were already scarce, and the number of referrals is now increasing. The use of digital interventions has increased during the COVID-19 pandemic, especially during physical distancing measures, and this has highlighted the increased role it can play in self-care and remote care. Digital interventions have the potential to improve the health outcomes of pregnant women and their experiences with care [29].

To our knowledge, no review has specifically focused on digitalized CBT interventions for depression symptoms during pregnancy. We note that 4 broader reviews on psychosocial interventions have been conducted in the past 5 years, and these can be divided into 2 categories. One review focused on any psychosocial interventions during pregnancy [30], and 3 reviews focused on psychosocial interventions throughout the broad perinatal period, namely pregnancy and the postnatal period [31-33]. The aim of the study by Li et al [30] was to carry out a systematic review and meta-analysis of any psychotherapies for pregnant women that focused on depression, anxiety, and quality of life. However, the authors only identified 2 RCTs on digitalized CBT. It was notable that their review did not include important search terms widely used for digital or internet interventions, such as web-based interventions, digital health, internet, and online interventions [34]. The other 3 reviews mainly identified interventions during the postnatal period. Lee et al [32] did not find any digitalized CBT interventions during pregnancy, while the other 2 studies [31,33] only found limited evidence. It is important to note that none of those 4 broader reviews contained extensive discussions of digitalized CBT interventions.

We decided that a new review was warranted, given the broad nature of previous systematic reviews and the increasing need for, and emergence of, digitalized CBT interventions for depression symptoms during pregnancy. Our systematic review aimed to evaluate the efficacy and acceptability of digitalized CBT interventions for depression symptoms during pregnancy. This review had 2 specific aims. The first was to evaluate the efficacy of digitalized CBT interventions during pregnancy by comparing the effect sizes between studies. The second was to assess the acceptability of digitalized CBT interventions by looking at attrition rates and feedback from participants.

## Methods

This systematic review was conducted in accordance with the PRISMA (Preferred Reporting Items of Systematic Reviews and Meta-analyses) [35] and the Synthesis Without Meta-analysis [36] reporting guidelines. The review protocol was prospectively registered with PROSPERO (the International Prospective Register of Systematic Reviews).

### Search Strategy

Relevant peer-reviewed papers published in English were identified by comprehensively searching key electronic databases from the earliest available evidence to October 27, 2021. These were the Web of Science, Cochrane Central Register of Controlled Trials, CINAHL, MEDLINE, Embase, PsycINFO, Scopus, ClinicalTrials.gov, and EBSCO Open Dissertations databases. The backward snowballing technique was also used to identify potentially relevant papers [37]. This involved looking at the reference lists of the selected papers to see if any more papers could be identified. The search strategy was created and refined based on consultation with a library information specialist (in Multimedia Appendix 1).

### Inclusion and Exclusion Criteria

The inclusion and exclusion criteria used to screen the articles were based on PICOS (population, intervention, comparator, outcome, and study design) [38].

#### Population

Studies were only included if the pregnant women were at least 18 years old. Studies were excluded if they combined samples of pregnant and nonpregnant women, male partners, or those aged younger than 18 years. Given that we focused on the broad spectrum of interventions for depression (prevention [universal, selective, indicated], treatment [case identification, standard treatment for known disorders], and maintenance [compliance with long-term treatment, aftercare] [22]), this may include a population of pregnant women with different depression risk groups.

#### Intervention

We focused on digitalized CBT interventions that primarily used technological platforms to target depression, such as the internet, computers, mobile phones, and virtual reality. The term digitalized CBT was based on 2 previously established definitions. First, we used the umbrella term digital, as it covers the full spectrum of digital technology, such as the internet, electronic devices, and mobile phones [39]. Second, we referred to the definition of technology-empowered CBT outlined by Wolters et al [40]: “CBT-based interventions integrating technology varying from basic online bibliotherapy to online self-help therapy, therapist-supported computerized CBT, smartphone applications (apps), traditional CBT delivered via telephone or videoconferencing, and combinations of these forms.” We only included interventions that began during pregnancy, but some of them also provided follow-up support during the postnatal period. We included any depression symptom intervention across the Institute of Medicine’s spectrum of interventions for mental health based on the risk of the target population: prevention (universal, selective, indicated), treatment (case identification, standard treatment for known disorders), and maintenance (compliance with long-term treatment, aftercare) [22]. Both guided and nonguided interventions were included. We excluded studies on psychosocial interventions that began postnatally and those that primarily evaluated nondigitalized CBT interventions, such as face-to-face group interventions and those with telephone support but no digital element.

#### Comparisons

Studies that compared digitalized interventions with standard treatment, waiting lists for treatment, and secondary or other interventions were included.

#### Outcome

Studies were included if the primary outcome was at least one validated measure of symptoms of depression during the perinatal period.

#### Study Design

Our review only included RCTs, as this research design is considered to be the gold standard for studying the effectiveness of interventions. RCTs are also considered to be the cornerstone for evidence-based practice [41], as they can be used to inform policy and practice decisions [42]. Only studies with full texts published in English were included. Nonrandomized, quasi-experimental, or pure qualitative studies were excluded and so were research protocols and case studies.

### Study Selection and Retrieval Process

**Figure 1:**
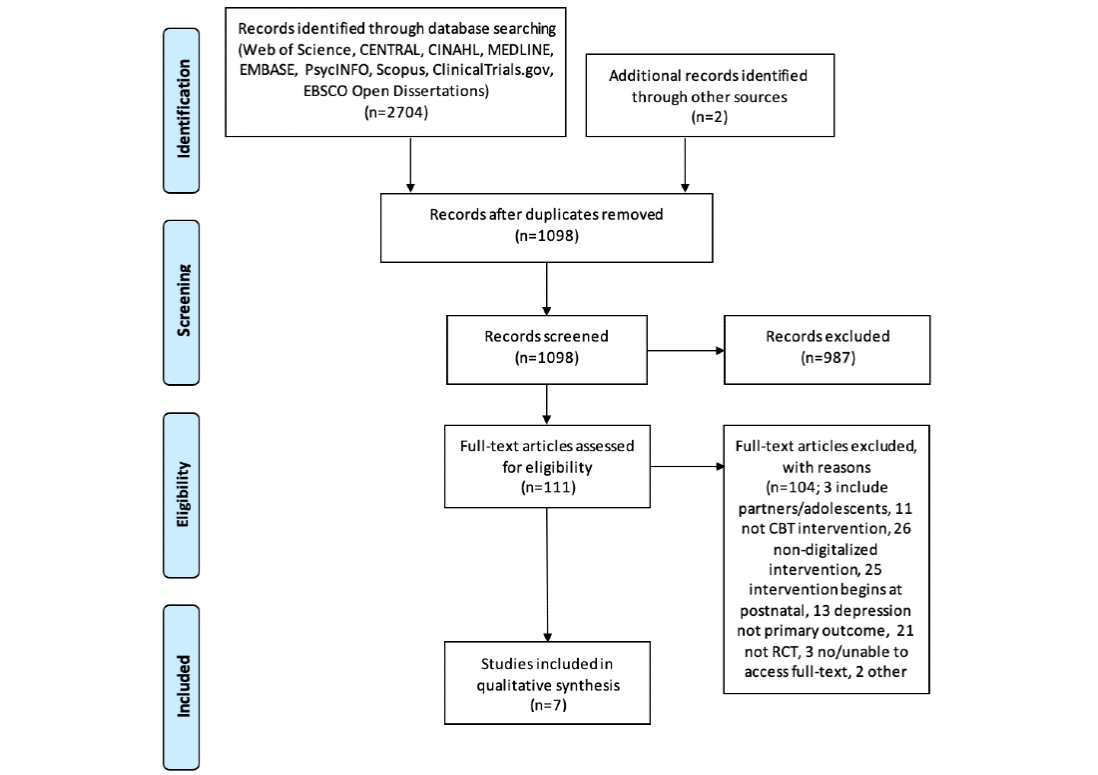
PRISMA (Preferred Reporting Items of Systematic Reviews and Meta-analyses) flow diagram.

Figure 1 displays the Preferred Reporting Items for Systematic Reviews and Meta-Analyses (PRISMA) flow diagram for the screening and study selection processes. The search and screening processes were independently conducted by 2 reviewers (WMAWMY and OW) based on the titles and abstracts and after any duplicates were removed. Any disagreements were discussed with a senior researcher (HMM). Next, the 2 reviewers independently conducted full-text assessments based on the predefined inclusion and exclusion criteria. Both reviewers cross-checked the papers that were suggested for inclusion, and any disagreements were discussed and resolved with the senior researcher (HMM) and professor (AS). The list of excluded studies with their reasons for exclusion are available in Multimedia Appendix 2.

### Quality and Risk of Bias Assessment

The methodological quality of the included studies was assessed using the Revised Cochrane risk-of-bias tool for randomized trials (ROB2) [43]. This tool addresses specific domains that can influence the risk of bias in an RCT. It asks a series of questions to enable users to evaluate the potential risk of bias in RCTs based on 5 domains: the randomization process, deviations from the intended intervention, missing outcome data, outcome measurements, and how the reported results were selected. The process evaluates each domain and enables the user to reach an overall judgement, namely a low or high risk of bias or some concerns. According to the tool guidelines, identifying a high risk of bias for any individual domain will produce an overall high risk of bias. Some concerns about any individual domain will lead to the paper being categorized as some concerns or a high overall risk [43]. The risk of bias assessments were independently conducted by 2 reviewers (WMAWMY and OW). Any discrepancies were discussed and agreed with the senior researcher (HMM) and professor (AS). The specific reasons for the 3 categorizations, namely low, some concerns, or high, were recorded in a Microsoft Excel spreadsheet template provided with the ROB2 tool.

### Data Extraction and Synthesis

A customized data extraction Excel spreadsheet was used by the reviewers. Data that were extracted from each of the final 7 papers included the author, year of publication, country, recruitment, sample size, and randomization. We also extracted the name, type, and mode of the intervention, screening method, primary depression scale, other mother-related outcomes, infant-related outcomes, number of sessions and duration, intervention start and end points, funding sources, brief synopsis, CBT elements, type of therapeutic guidance, and other support, such as technical or peer support. The research team also extracted and calculated the between-group effect sizes and 95% CIs after the intervention, user feedback, and the postintervention attrition rates. The Hedges g effect sizes were either extracted, if provided, or calculated using the 2020 Effect Size Calculator from the Center for Evaluation and Monitoring [44]. We extracted the means, standard deviations, and sample sizes of the intervention and control groups at the postintervention assessment point, during pregnancy. The effect sizes were interpreted as small (≤0.32), moderate (0.33 to 0.55), or large (≥0.56) [45]. The acceptability of the interventions was assessed according to the Theoretical Framework of Acceptability [46]. This suggests that intervention acceptability can be assessed at 3 time points: before, during, and after an intervention has been delivered [46]. In addition, the attrition rate percentage was calculated using the total number of subjects dropped out in the intervention group post-intervention over the total number of subjects in the intervention groups at baseline, after randomization. The data extraction was conducted by the first author (WMAWMY) and then independently checked by the second reviewer (OW) for accuracy and completeness. Disagreements were resolved through consensus discussion with a senior researcher (HMM). WMAWMY also contacted authors of included studies with a request for missing or additional information (in Multimedia Appendix 3).

## Results

The electronic searches and hand searches yielded 2706 titles, and 7 were included in this study after the duplicates were removed and the screening process was carried out [47-53]. All of the papers were published between 2015 and 2021, and 5 were published in 2019 or 2021. Figure 1 provides a flow diagram of the screening processes and study selection.

### Characteristics of the Included Studies

Table 1 summarizes the characteristics of the 7 studies: 2 were from the United States, 2 were from China, and there was 1 each from Australia, Norway, and Sweden. The papers covered a total of 2830 participants, and the sample sizes ranged from 25 to 1342 at the time of randomization. The 7 studies assessed 7 different interventions. The Swedish Internet Cognitive Behavior Therapy for Antenatal Depression program had 42 participants, and the intervention, which was delivered via a secure online platform, consisted of reading material, assessments, homework, and worksheets [49]. Videos, graphics, and an online diary book were used by the Chinese Internet-based Mindful Self-Compassion Program with 314 participants [50]. The Mothers and Babies Internet Course had 852 participants, making it the second largest study. Coordinated from the United States, but with a worldwide reach, it provided fully automated online lessons that included information pages, short audio and video clips, images, and worksheets [47]. The American-based Sunnyside Group-based Internet Intervention was an online website with various interactive tools and games that facilitated learning and peer interaction. It was the smallest study, with 25 participants [48]. Mamma Mia was by far the biggest program, with 1342 Norwegian participants. It was a fully web-based intervention that combined text, pictures, pre-recorded audio files, and user input [51]. The Australian MUMentum Pregnancy Program had 87 participants. This featured a virtual online clinic and used an illustrated story centered around fictional characters [52]. The final study was the Chinese smartphone-based Mindfulness Behavioral Cognitive Therapy, with 168 participants, delivered through a smartphone app. It consisted of thematic curriculum provided through text, audio, and visual materials [53].

Two of the studies, from the United States [47] and Norway [51], employed a universal approach, and the other 5 studies used an indicated approach. Three studies recruited participants directly from hospitals: the Chinese study from a reproductive mental health multidisciplinary clinic [50], another Chinese study from the obstetrics clinic of a tertiary hospital [53], and the largest Norwegian study via midwives during regular maternal check-ups [51]. The other 4 studies combined diverse recruitment strategies, using advertisements on social media and online forums and in newspapers, mass emails, Google Ads, and flyers distributed at maternity clinics and hospitals. None of the studies screened participants using population-based approaches. Four studies screened participants during recruitment using different measures and cut-off scores. The Swedish study used a Montgomery-Åsberg Depression Rating Scale (MADRS) [54] score of 5 to 35 and no or a low risk of suicide [49]. The Chinese study used an Edinburgh Postnatal Depression Scale (EPDS) [55] score of ≥9 [50], while the other Chinese study used an EPDS score of ≥9 or Patient Health Questionnaire (PHQ)-9 [56] of ≥4 [53]. The small American study used PHQ-8 scores of 5 to 14, which indicated no diagnosis of major depression [48]. When it came to measuring depression, 6 studies used the EPDS, as either the only measure or one of the outcome measures. However, the EPDS outcome cut-off scores used for probable depression differed: 4 studies used >9 points [47,50,51,53], and 2 studies used >12 points [49,52]. Other studies used the PHQ-9, Center for Epidemiological Studies-Depression [57], MADRS-Self Report, and Beck Depression Inventory II [58] to measure depression outcomes. The studies also assessed various other mother-related outcomes, but only 2 studies reported infant-related or mother-infant attachment outcomes. These were the Chinese study [50], which used the Infant Behavior Questionnaire Revised-Very Short Form [59] and the Australian study [52], which used the Maternal Antenatal Attachment Scale [60]. Five studies [48-50,52,53] also assessed anxiety as secondary outcomes, using the Generalized Anxiety Disorder 7 scale [61], the State-Trait Anxiety Inventory [62], or the Inventory of Depression and Anxiety Symptoms [63].

**Table 1:** Summary of included studies.

Author, reference and country			Recruitment		Intervention name		Randomization and sample size		Intervention type and mode		Screening		Primary depression scale		Other mother-related outcomes		Infant-related outcomes
**Guided**																	
	Forsell et al [49], Sweden	Advertisements on social media, blogs, online forums, in newspapers, and flyers distributed in maternity clinics		Internet Cognitive Behavior Therapy for Antenatal Depression		Intervention: n=22; controls: n=20; total: n=42		Indicated, internet and website for intervention group with treatment as usual for controls		MADRS-S^a^ scores of 15 to 35 and no/low risk of suicide		MADRS-S		EPDS^b^ (>12), WSAS^c^, GAD^d^, ISI^e^, EQ-5D-3L^f^, AUDIT^g^, DUDIT^h^		-^i^	
	Guo et al [50], China	Pregnant women attending Tianjin First Center Hospital		Internet-based Mindful Self-Compassion Program		Intervention: n=157; controls: n=157; total: n=314		Indicated, internet and website for intervention group with waiting-list control group		EPDS (≥9)		EPDS (>9)		STAI^j^-I and II, BDI^k^-II, Chinese Mindfulness Attention Awareness Self-Compassion Scale, WHO-5^l^, PSI^m^, Comprehensive Parenting Behavior Questionnaire		IBQ-R VSF^n^	
**Unguided**																	
	Barrera et al [47], United States	Search engine directories and Google Ads Worldwide reach		Mothers and Babies Internet Course		Intervention: n=435; controls n=417; total: n=852		Universal, internet, and website for intervention group with information only for controls		N/A^o^		CESD^p^		EPDS (>9), Major Depressive Episode Screener		-	
	Duffecy et al [48], United States	Pregnant women invited after being identified from electronic records of a university hospital; mass emails and advertisements		Sunnyside Group-based Internet Intervention		Intervention: n=18; controls: n=7; total: n=25		Indicated, internet and website for intervention group with extra features for intervention group and just online group for controls		PHQ^q^ scores of 5 -14 and no diagnosis of major depression		PHQ-8		HDRS^r^, IDAS^s^, SCID-I^t^, MINI^u^ Suicide		-	
	Haga et al [51], Norway	Women attending routine prenatal care in hospital clinics		Mamma Mia		Intervention: n= 678; controls: n=664; total: n=1342		Universal, internet and website for intervention group, with usual perinatal care for controls		N/A		EPDS (>9)		-		-	
	Loughnan et al [52], Australia	Advertisements on social media, online forums, and flyers distributed in maternity hospitals		MUMentum Pregnancy Program		Intervention: n=43; controls: n=44; total: n=87		Indicated, internet and website for intervention group, with treatment as usual for controls		Met the criteria for a probable diagnosis of generalized anxiety disorder and/or major depressive disorder		PHQ-9		GAD-7, Kessler 10-item Psychological Distress scale, EPDS (>12), WHO-QOL^v^, 9-item BDI-II		MAAS^w^	
	Sun et al [53], China	Obstetrics clinic of a tertiary hospital in Jinan, Shandong		Spirits Healing (in Chinese) app		Intervention: n=84; controls: n=84; total: n=168		Indicated, internet and smartphone app for intervention group, with regular WeChat health consultations for controls		EPDS (≥9) or PHQ-9 (≥4)		EPDS		GAD-7, PSS^x^, PANAS^y^, PSQI^z^, PRMQ^aa^, W-DEQ^ab^		-	

^a^MADRS-S: Montgomery-Åsberg Depression Rating Scale-Self report version.

^b^EPDS: Edinburgh Postnatal Depression Scale.

^c^WSAS: Work and Social Adjustment Scale.

^d^GAD: Generalized Anxiety Disorder.

^e^ISI: The Insomnia Severity Index.

^f^EQ-5D-3L: EuroQoL 5-Dimension 3-Level.

^g^AUDIT: Alcohol Use Disorders Identification Test.

^h^DUDIT: Drug Use Disorders Identification Test.

^i^None reported.

^j^STAI: State-Trait Anxiety Inventory.

^k^BDI: Beck Depression Inventory.

^l^WHO-5: World Health Organization 5 Well-Being Index.

^m^PSI: Parenting Stress Index.

^n^IBQ-R VSF: Infant Behavior Questionnaire Revised-Very Short Form.

^o^N/A: not applicable.

^p^CESD: Center for Epidemiological Studies-Depression.

^q^PHQ: Patient Health Questionnaire.

^r^HADRS: Hamilton Depression Rating Scale.

^s^IDAS: Inventory of Depression and Anxiety Symptoms.

^t^SCID-I: Structured Clinical Interview for Diagnostic and Statistical Manual of Mental Disorders Axis-I Disorders.

^u^MINI: Mini International Neuropsychiatric Interview.

^v^WHO-QOL: World Health Organization Quality of Life scale.

^w^MAAS: Maternal Antenatal Attachment Scale.

^x^PSS: Perceived Stress Scale.

^y^PANAS: Positive and Negative Affect Schedule.

^z^PSQI: Pittsburgh Sleep Quality Index.

^aa^PRMQ: Prospective and Retrospective Memory Questionnaire.

^ab^W-DEQ: Wijma Delivery Expectancy Questionnaire.

### Interventions Used by the Studies

**Figure 2:**
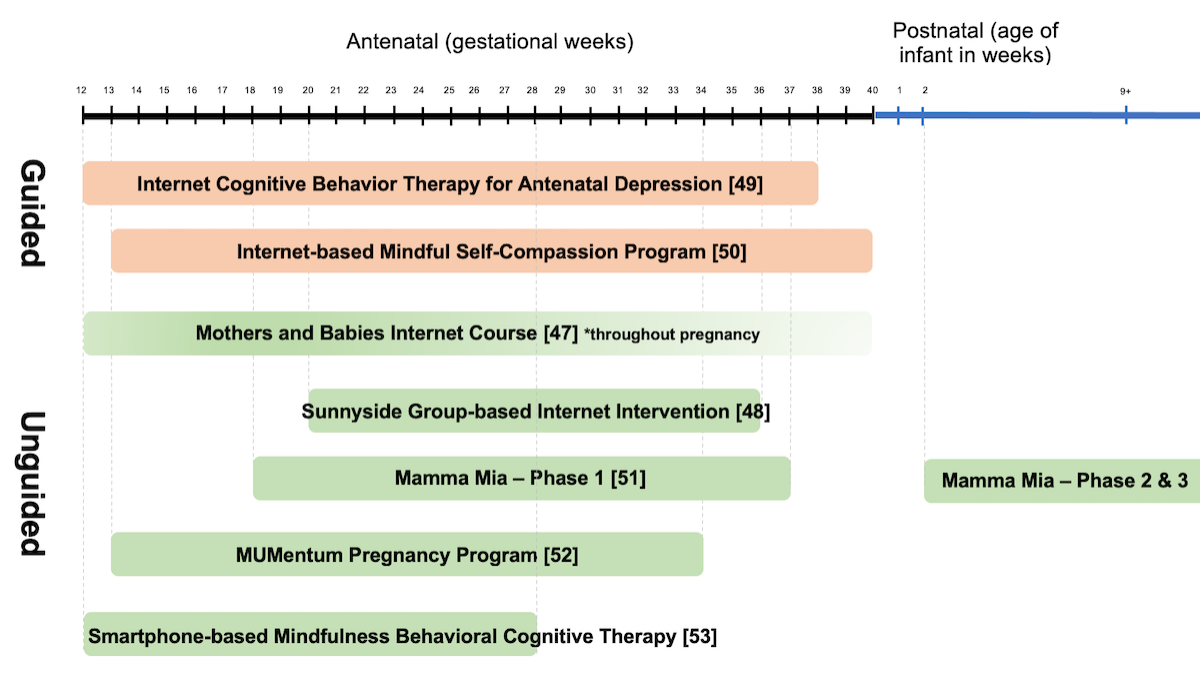
Comparison of interventions from the earliest starting point to the latest endpoint.

Table 2 summarizes the description of each intervention, while Figure 2 compares the earliest starting point to the latest end point for each intervention. All the digitalized study interventions were delivered on the internet but varied in terms of their session structure and duration, the type of support provided, and the duration. Six interventions were delivered via a website, and only 1 [53], which was recently published, was delivered via a smartphone app. The shortest intervention, from Australia, lasted for 4 weeks and was comprised of 3 brief unguided internet-based CBT (iCBT) sessions [52], while the largest Norwegian intervention, Mamma Mia, had the longest duration, of 11.5 months, and provided sessions during pregnancy and postnatal periods [51]. Two of the 7 interventions were guided, as they incorporated an element of therapeutic guidance. The Swedish intervention was provided by CBT-trained therapists, who were not mental health professionals, on a fully digital platform [49], but no telephone guidance was provided. The level of therapeutic guidance provided by this guided iCBT varied, and messages sent via the internet platform were responded to within 48 hours during weekdays. The Chinese intervention did not detail the type of guided instructions that were provided [50]. In addition to therapeutic guidance, several of the guided and unguided interventions employed other forms of support, such as peer support discussion groups or platforms, as well as technical support by the research team members. Table 3 shows a brief synopsis and the CBT elements of the 7 interventions.

**Table 2:** Summary of the interventions in the included studies.

Author(s) and country			Intervention name		Age (years), mean (SD), range		Type of therapeutic guidance		Other support (eg, technical or peer)		Funder
**Guided**											
	Forsell et al [49], Sweden	Internet Cognitive Behavior Therapy for Antenatal Depression		Intervention: 31.2 (3.7); controls: 30.8 (5.3)		Personalized feedback using written online messages. Therapists only had basic CBT^a^ training and no prior experience nor any special education or training in order to treat this specific population		Anonymous online discussions between participants		Swedish Research Council, regional agreement between Karolinska Institutet and Stockholm City Council, and regional agreement between Umeå University and Västerbotten County Council (ALF)	
	Guo et al [50], China	Internet-based Mindful Self-Compassion Program		Intervention: 29.8 (6.2); controls: 31.4 (5.7)		Detailed information not available		No information provided		No information provided	
**Unguided**											
	Barrera et al [47], United States	The Mothers and Babies Internet		Intervention: 29.81 (6.09), 18-43; controls: 30.59 (4.99), 19-42		N/A^b^		No information provided		National Institute of Mental Health, Robert Wood Johnson Health Disparities Seed Grant, University of California Committee on Latino Research, and SFGH^c^ Department of Psychiatry	
	Duffecy et al [48], United States	Sunnyside Group-based Internet Intervention		Intervention: 30.5 (4.05), 25-45; controls: not provided		N/A		Peer support and contact moderator tool for technical or group issues		NIMH, National Center for Advancing Translational Sciences of the NIH^d^	
	Haga et al [51], Norway	Mamma Mia		Intervention: 31.0 (4.6); controls: 31.1 (4.5)		N/A		Notification to talk to someone or seek professional help when the presence of some or many depression symptoms		Research Council of Norway	
	Loughnan et al [52], Australia	MUMentum Pregnancy Program		Intervention: 31.69 (4.44); control: 31.54 (3.63)		N/A		Technical assistance by research technicians		NHMRC^e^, HCF^f^ Research Foundation, Rotary Health Australia, and the David Henning Memorial Foundation	
	Sun et al [53], China	Spirits Healing (in Chinese) app		Intervention: 30.27 (3.80); control: (29.55) 4.21		N/A		No information provided		Chinese National Funding of Social Sciences and China Scholarship Council	

^a^CBT: cognitive behavioral therapy.

^b^N/A: not applicable.

^c^SFGH: San Francisco General Hospital.

^d^NIH: National Institutes of Health.

^e^NHMRC: National Health and Medical Research Council.

^f^HCF: Hospitals Contribution Fund.

**Table 3:** Brief intervention synopsis, themes, and cognitive behavioral therapy (CBT) elements.

Author(s) and country			Brief synopsis		Themes		CBT elements
**Guided**							
	Forsell et al [49], Sweden	The iCBT^a^ Internet Psychiatry Clinic is an intervention for antenatal depression and is an adapted version of iCBT for depression. The platform is a form of guided self-help treatment consisting of reading material (about 75,000 words), assessments, homework, and worksheets via a secure online platform. The platform can be accessed anytime and anywhere using a computer or mobile device with an internet connection. There are 10 modules of guided self-help by nonexpert therapists trained in CBT.		Introduction, being pregnant, behavioral activation, cognitive restructuring, relationships, anxiety and worry, sleep problems, and summary and relapse prevention		Psychoeducation (depression, CBT, myths, facts, and physiological changes), behavioral activation (positive and negative reinforcement behaviors), cognitive restructuring (negative automatic thoughts, acceptance, cognitive biases), psychoeducation (relationships, communication, role transition, anxiety and fear of labor and sleep), and homework	
	Guo et al [50], China	Mindful Self-Compassion Program (MBSP) is aimed at promoting self-regulatory skills of pregnant women at high risk for postpartum depression focusing on self-compassion. The program utilizes videos involving different types of exercises with guided instructions sequentially provided after completion of a previous module. The 6-week program lasts 10 hours with guided instructions: 36 episodes each lasting about 15 minutes		Not available		Largely based on mindfulness CBT; understanding and applying self-compassion, skills to manage difficult emotions rather than solving specific problems, exercises with guided instructions. Users were encouraged to practice the skills during the day and provided with an online diary book for reflection.	
**Unguided**							
	Barrera et al [47], United States	The Mothers and Babies Internet (e-MB) is aimed at Spanish- and English-speaking pregnant women to reduce the risk of postpartum depression. The e-MB consists of fully automated lessons and is sequential in nature, whereby each session needed to be completed first before proceeding to the next lesson. Following completion of each session, participants may access the lesson and worksheets infinitely for review. The e-MB consisted of 8 flexible sessions of fully automated self-help.		Not available		Information pages, audio, video, images, and worksheets based on the cognitive behavior framework, social learning theory, reality management training, attachment theory, and diverse sociocultural issues	
	Duffecy et al [48], United States	The Sunnyside website group intervention consisted of 10–15-minute didactic lessons using text and video material and included several interactive features in the form of (1) an activity feed, which was a constantly updating feed that displayed each of the women’s activity on the site, whereby other participants can like and comment or provide feedback to other women’s posts, (2) discussion questions posted by study staff after each session to encourage interaction, (3) individual garden plot and community garden linked to individual’s profiles and providing gamification and interactive features individually and between other users, (4) contact moderator tool to report any issues with the site or with group members. After each session, participants are prompted with a “call to action” encouraging them to apply the skills learned during the sessions. The intervention consisted of an 8-week unguided online program.		Your mood and your pregnancy, worries about me and my baby, mood management, challenging your thinking, positive activity during pregnancy, physical activity during pregnancy, partner communication and support, body image and sex during pregnancy and postpartum, relationships with your mother and mother-in-law, challenges in relationships with friends and others, monitoring kick counts and other pregnancy anxiety, anxiety and parenthood, relaxation, employment issues, during and after the birth, moving forward, and conclusions		Psychoeducation, mood management, thought challenging, positive and physical activities, relationship with partner and others in the social circle, anxiety during pregnancy and parenthood, relaxation, employment issues and managing resources during and after labor; 5 interactive CBT tools: thought restructuring (think), mood tracking (feel), activity scheduling and monitoring (do), relaxation (relax), and goal setting (achieve)	
	Haga et al [51], Norway	Mamma Mia is a free universal preventive intervention for perinatal depression. The intervention is delivered by email and interactive websites, combining text, pictures, prerecorded audio files, and user input. User receives an email with a hyperlink for each session that lasts around 10 minutes each. The hyperlink directs the user to the Mamma Mia web page, and the intervention content proceeds sequentially to the next web pages (tunnel information architecture) to ensure continuity of the program narrative. The intervention consists of 3 fully automated phases with 44 sessions over 11.5 months: phase 1 (during pregnancy); phase 2 (infant is 2-3 weeks old, for 6 weeks); and final phase (10 sessions over an 18-week period)		Knowledge, expectancies and attitudes, attachment, emotion regulation, and help-seeking, relationship satisfaction, and communication skills		Assessment of depressive symptoms, metacognitive therapy, positive psychology, couples’ therapy, breastfeeding, and psychoeducation; the metacognitive element emphasized the process of inflexible and recurrent thinking style due to negative thoughts, feelings, or beliefs. Acceptance commitment therapy and mindfulness elements were also incorporated.	
	Loughnan et al [52], Australia	The MUMentum Pregnancy transdiagnostic intervention targets anxiety and depression symptoms and is delivered via the online Virtual Clinic system. The program emphasized a short, illustrated story centered around 2 fictional characters experiencing depression and anxiety during pregnancy. The characters learn to manage their symptoms by applying CBT skills in the context of the character experiences, challenges, and symptoms common during pregnancy. The system employs a 7-day lockout period implemented between lessons to ensure participants spend time revising and implementing the lesson material before moving onto the next lesson. Of this, participants are also notified via email and SMS reminders regarding new lessons and to stay on schedule. The 4-week program consists of 3 brief unguided self-help lessons with only technical support.		About anxiety and depression, identifying symptoms, cognitive behavioral model, prioritizing self-care, physical symptoms, partners and supporters, controlled breathing, progressive muscle relaxation, about thoughts, identifying unhelpful thoughts, shifting unhelpful thoughts, accepting uncertainty, thought challenging, coping cards, structured problem-solving, unhelpful behaviors (low activity; avoidance), facing your fears, activity planning and monitoring, graded exposure, assertive communication, relapse prevention		Transdiagnostic intervention for depression and anxiety during pregnancy; involved psychoeducation, cognitive restructuring, problem-solving, behavioral activation, and relapse prevention; each lesson illustrated the characters’ stories: introduction to core CBT skills, summary, and action plan to implement the skills and several supplementary resources.	
	Sun et al [53], China	The Spirits Healing (in Chinese) app is a mindfulness training program for use during pregnancy for perinatal depression and other mental health problems. It was available for Android and iOS operating systems in mainland China. The app provides reading materials, recordings for guided practice, videos, and a mindfulness journal that can be accessed anytime and utilized at users’ own pace. Weekly reminder messages were sent via WeChat for users to complete the training. Participants were awarded 2 yuan (US $0.30) for completion of each week of training or each completion of assessment. The 8-week mindfulness CBT training automatically updated every day, and participants practiced according to their own schedules.		Understand mindfulness, be in the present, be mindful of negative emotions, accept difficulties, thoughts are just thoughts, enjoy daily happiness, mindful pregnancy and childbirth, continued mindfulness practice		Following psychoeducation information via the app, formal mindfulness training techniques were introduced. Users are encouraged to continue to practice, and they are supplemented with recordings and a mindfulness journal. Formal training included body scan, mindful breathing, mindful stretching, and mindful meditation. Informal training included encouragement to practice every day, pausing in the midst of daily life, mindful eating, mindful walking, and 3-minute breathing practices.	

^a^iCBT: internet-based CBT.

### Assessment of Risk of Bias

**Figure 3:**
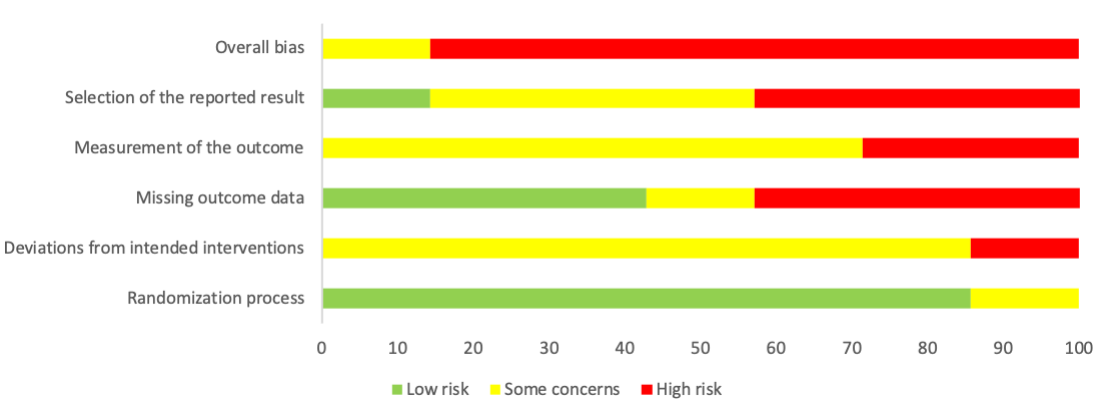
Percentage of risk of bias based on the 5 Revised Cochrane risk-of-bias tool for randomized trials (ROB2) domains.

**Figure 4:**
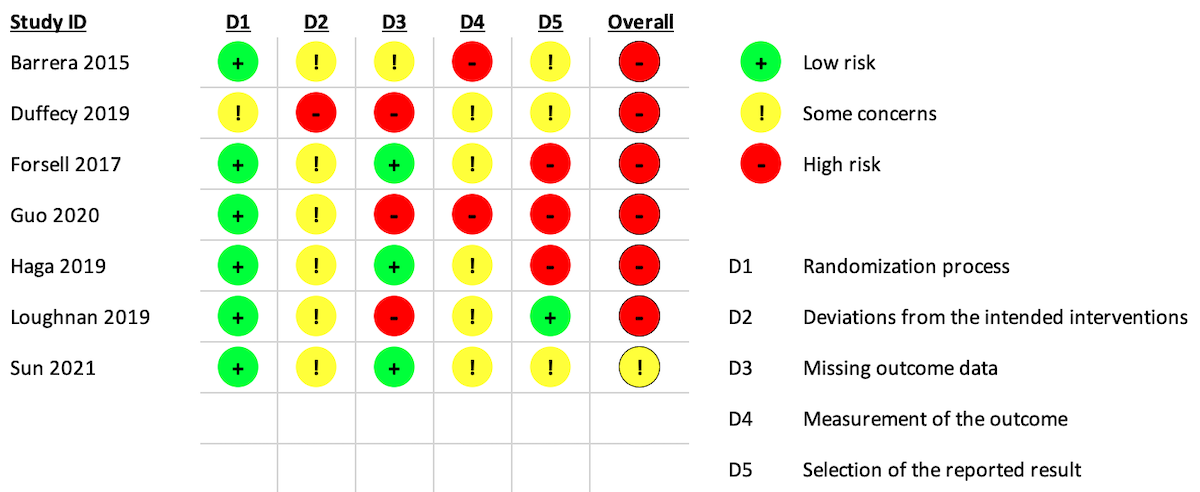
Risk of bias for depression outcomes, based on the individual studies.

Figure 3 and Figure 4 display the risk of bias assessment results for each study. Six studies had a high overall risk of bias, as a high risk of bias was recorded in at least one domain, while 1 study recorded some concern for overall risk of bias. Self-reported measures are commonly used to evaluate psychological outcomes, but there were at least some concerns about the risk of bias that these provided in 7 studies, based on the ROB2 guideline [43]. The missing outcome data domain was of some concern, given nearly half (3/7, 43%) of the studies did not provide sufficient information about the reasons for dropout between groups or analyses to address these missing data. In addition, the way that the reported results were selected was of high concern. These were particularly related to the fact that 5 of the study protocols were not registered or there were inconsistencies, without justification, between the registered protocols and the published papers. These inconsistencies included the absence of follow-up results in the published paper or differences between these and the intended analysis plan in the published protocol.

### Efficacy of Digitalized CBT Interventions for Depression Symptoms During Pregnancy

**Figure 5:**
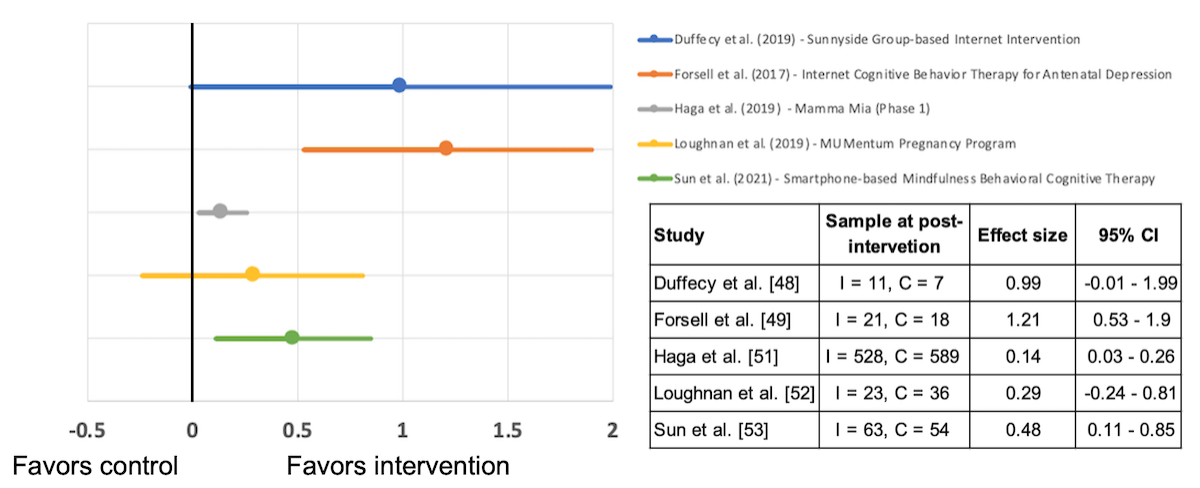
Hedges g effect sizes (95% CIs) for depression after programs in the intervention (I) and control (C) groups.

In view of the overall high risk of bias in all except one of the studies, the efficacy of the interventions needs to be interpreted with caution. Five studies provided sufficient data for effect size calculations. Figure 5 summarizes the Hedges g effect size for the 5 studies [48,49,51-53]. In general, the more positive results for the intervention than for the control groups showed promising evidence for the efficacy of digitalized CBT interventions for pregnant women. It was notable that there were differences in efficacy between the guided and unguided interventions. The highest postintervention effect size was recorded for the Swedish Internet Cognitive Behavior Therapy for Antenatal Depression program, which focused on pregnant women who were judged to have no or a low risk of suicide when screened for symptoms of depression [49]. The remaining 4 interventions were all unguided and recorded large [48], moderate [53], or small [51,52] effect sizes.

### Acceptability of Digitalized CBT Interventions

Table 4 compares the acceptability assessments conducted by individual studies based on 3 time points: before, during, and after the intervention was delivered. Overall, most of the participants were satisfied with the interventions they received, but high attrition rates were recorded for the 4 unguided interventions (22.1%-46.5%). The way that intervention acceptability was measured varied significantly between the studies, especially the scales that were chosen. All the studies obtained feedback after the interventions had finished. Five studies assessed acceptability during the interventions [48,49,51-53], while only 2 studies assessed acceptability before they were used [48,51]. The highest attrition rates for the unguided interventions were for the unguided MUMentum Pregnancy transdiagnostic intervention [52]. In contrast, the guided intervention recorded a very low attrition rate of 4.5% for the Swedish Internet Cognitive Behavior Therapy for Antenatal Depression program [49].

**Table 4:** Acceptability before, during, and after the interventions.

Author(s) and country		Acceptability before	Acceptability during	Acceptability after	Attrition
**Guided**					
	Forsell et al [49], Sweden	N/A^a^	Treatment Credibility Scale of the Credibility/Expectancy questionnaire: good treatment credibility	Client Satisfaction Questionnaire 8: good satisfaction level; treatment adherence and utilization described	4.5%
	Guo et al [50], China	N/A	N/A	Brief dropout reasons provided; attendance rate=91.8%	N/A
**Unguided**					
	Barrera et al [47], United States	N/A	N/A	3 open-ended questions: intervention helpfulness and usefulness rated favorably; content easy to understand	N/A
	Duffecy et al [48], United States	Intervention development process involved target participants; topics, site motif (visual themes and look and feel of the internet site), and usability of potential application	Use of interactive features assessed	Usability, satisfaction, and ease of use: intervention usefulness, ease of use, ease of learning, satisfaction rated favorably	38.9%
	Haga et al [51], Norway	Intervention development process published in Drozd et al [64]	Dropout reasons not described in paper; other acceptability and feasibility details in paper [65]	More than half completed >80% of intervention; other acceptability and feasibility details in the paper [51]	22.1%
	Loughnan et al [52], Australia	N/A	Detailed dropout reasons provided; intervention content evaluated during each session	Treatment Satisfaction Questionnaire: high satisfaction; Credibility and Expectancy Questionnaire: intervention quality rated as excellent; Intervention utilization and implementation data provided	46.5%
	Sun et al [53], China	N/A	Logs of practice on formal mindfulness training	Completion rates for all 8 sessions=8.3%; completion rates for 4 sessions=52.4%	25%

^a^N/A: not available.

## Discussion

### Principal Findings

Our review identified 7 RCTs of digitalized CBT interventions for depression symptoms during pregnancy that were promisingly efficacious and had good acceptability. RCTs using digital CBT for depression during pregnancy have been scarce in contrast to the number of studies on digitalized CBT interventions for depression overall. We judged 6 of the 7 studies to have a high risk of bias. The high risk of bias was mainly due to missing outcome data and selection of reported results domains. These point to the need to provide accessible trial registration information with sufficient information prior to commencement of controlled trials. Although missing data in psychological intervention studies are common, a detailed description of how these missing data were addressed and reasons for dropouts in the intervention and control group need to be clarified and compared in each study. Therefore, the findings on efficacy should be considered with caution, and more high-quality studies are needed. Despite these limitations, this review produced 3 main findings. First, technology can be used to deliver CBT programs that target depression during pregnancy. Second, digitalized CBT during pregnancy showed promising evidence of efficacy and positive outcomes for depression (Hedges g for guided interventions=1.21; Hedges g for unguided interventions=0.14-0.99). Third, the digitalized CBT interventions were well-received by pregnant women, especially interventions that were guided, which had lower attrition rates than the unguided interventions (4.5% versus 22.1%-46.5%).

CBT interventions have traditionally been delivered face-to-face, but technological advances have revolutionized the way they are being adapted to various platforms. The COVID-19 pandemic has also led to an urgent increase in the need for digital care delivery. For the past 20 years, the progress in digitalized interventions has been rapid, and various evidence-based digitalized interventions have been developed and disseminated [66], especially for depression [67]. However, little is known about digitalized CBT interventions for depression during pregnancy. All 7 interventions identified in this review used the internet and website or smartphone app platforms to deliver core elements of CBT, such as psychoeducation, cognitive restructuring, problem-solving, and behavioral activation. The central themes of these were managing challenges and expectations during pregnancy by using CBT skills. These platforms contained a combination of text, images, and videos to make the interventions attractive and engaging. Other additional interactive features were unique to some interventions. For example, the American-based Sunnyside Group-based Internet Intervention included an activity feed that displayed each participant’s activity on the site. This meant that other participants could like, comment, or provide feedback on other women’s public posts. It also had an individual garden plot and community garden that were linked to individual profiles. The intervention provided individual and group games, where garden gnomes or flowers could be collected by completing various tasks [48]. Likewise, the Swedish Internet Cognitive Behavior Therapy for Antenatal Depression program provided an online discussion group, in which participants could interact with each other anonymously [49]. These unique interactive features allowed participants to share their lived experiences during pregnancy, as well as provided a platform for emotional peer support. These are both considered to be key functions of traditional online pregnancy forums [68].

The limited evidence and high risk of bias in 6 out of the 7 papers we reviewed prevented us from coming to firm conclusions on the efficacy of digitalized CBT for depression during pregnancy. However, our review does provide promising evidence on the efficacy and positive impact of digitalized CBT for pregnant women. Previous research has reported that the prevalence of postnatal depression symptoms can be predicted by depression symptoms during pregnancy [3]. Interventions during pregnancy allow participants to acquire and apply coping skills at an early stage, potentially before their depressive symptoms worsen. It should also be noted that 5 of the included studies used standard care, an active control, or another intervention as control groups, which may partly explain the modest effect sizes in most of the studies. The Swedish intervention [49] had an exceptionally high effect size. Unsurprisingly, that intervention used a guided and indicated approach. The intervention was delivered via an online internet platform comprised of about 75,000 words of reading material, assessments, homework, case stories, and worksheets. The therapists provided regular individual feedback, encouragement, and support in written messages, with replies within 48 hours on weekdays. Interestingly, the therapeutic guidance was provided by CBT-trained therapists who were not mental health professionals. Compelling evidence from systematic reviews and meta-analyses of iCBT research for depression has highlighted the importance of providing support from a therapist or human guidance for effective interventions [69-72].

Six out of 7 studies we reviewed also assessed other self-reported maternal outcomes: anxiety, psychological distress, alcohol and drug use, mindfulness attention awareness, general health, stress, self-compassion, well-being, quality of life and work and social adjustment, positive and negative affect, sleep-related problems, fatigue, prospective memory, retrospective memory, and fear of childbirth. Only one study assessed infant outcomes, using the Infant Behavior Questionnaire, and another study assessed mother-child attachment or bonding using the Maternal Antenatal Attachment Scale. This was somewhat surprising, as there is compelling evidence that prenatal depression has been associated with various infant-related issues, including temperament, bonding, emotional and behavioral problems, cognitive impairment, and psychopathology [73-79]. The fact that there was so little research on infant-related or mother-infant attachment outcomes by the studies we reviewed limits any insightful discussion and highlights the need for further evaluation.

All the studies we reviewed reported diverse acceptability outcomes for at least 1 of the 3 time points: before, during, and after the intervention was delivered. All the interventions recorded good acceptability, especially after the intervention had been completed, as the participants were satisfied with the interventions and felt that they were useful. Acceptability data before and during intervention delivery are still lacking, and more acceptability components can be explored further. The Theoretical Framework of Acceptability outlines 7 components of acceptability that could be explored in intervention studies at these 3 time points: affective attitude, burden, perceived effectiveness, ethicality, intervention coherence, opportunity costs, and self-efficacy [46]. Including all the acceptability data in a single paper may be challenging due to word count limits or information overload. To deal with this issue, authors could provide acceptability evaluations in separate papers. For example, the Mamma Mia intervention included in our review was the focus of 3 separate papers on intervention development [64], acceptability and feasibility [65], and efficacy [51].

The attrition rates for the unguided interventions were high (22.1%-46.5%), and this finding was consistent with previous meta-analyses on digitalized CBT interventions for depression [70,72]. The terms “universal” and “indicated” were based on the Institute of Medicine’s spectrum of mental health interventions based on the risk for the target population: prevention (universal, selective, indicated), treatment (case identification, standard treatment for known disorders), and maintenance (compliance with long-term treatment, aftercare) [22]. Our review identified 2 “universal” interventions (developed for pregnant women with any risk level for diagnosable clinical depression) and 5 “indicated” interventions (developed for pregnant women already at risk for diagnosable clinical depression [eg, those that exceed a cut-off screening score]). These classifications (ie, universal, indicated) have also been used in other perinatal distress, depression, and anxiety research [80].

Guided versus unguided and universal versus indicated approaches may have their own merits when it comes to impact. For instance, the study on the unguided and universal Mamma Mia intervention recruited the highest number of participants, 1342, with 678 in the intervention group and 664 receiving standard care. Although this study showed small effect sizes, partly due to its universal approach, more than one-half (345/678, 50.9%) of the total participants in the intervention group completed 80% or more of the year-long program, with the first phase during pregnancy and the second and third phases during the postnatal period. Any approach requires consideration based on the readiness of pregnant women to change their health behavior, particularly with reference to the Capability, Opportunity, Motivation, Behavior (COM-B) framework [81]. In general, pregnancy has been called a “teachable moment,” which is described by Phelan [82] as naturally occurring life transitions or health events that are thought to motivate individuals to spontaneously adopt risk-reducing health behaviors. Although pregnancy is a time when women may benefit from increased motivation for behavioral change, based on the COM-B framework, such changes can be difficult to realize if their capabilities and opportunities are neglected [83,84]. As discussed, earlier digitalized CBT may provide the opportunity for pregnant women to access digitalized interventions. However, their lack of capacity, namely reduced energy, may prevent the women from continuously engaging with the program. That is why it is crucial for those who develop interventions to consider the 3 elements of capacity, opportunity, and motivation together, in order to minimize attrition.

Pregnancy is a relatively short period, and shorter interventions could attract pregnant women to engage. CBT is problem focused and based on the way that people think and behave, and technology can make its delivery much more cost-effective. This was proven by the Australian MUMentum Pregnancy Program included in our review [52]. The transdiagnostic intervention targeted both depression and anxiety, by using a story that centered around 2 fictional characters experiencing depression and anxiety during pregnancy. The characters learned to use CBT skills to manage their symptoms, based on their experiences and challenges during pregnancy. The program received highly positive feedback from participants, in terms of satisfaction, quality, and how they became immersed in the storyline. The small effect size recorded by this unguided intervention should not mask its potential benefits. It should be emphasized that, during the study period, participants in the treatment-as-usual control group continued to receive existing health care services. This might have had a confounding impact on their symptom levels, compared with the MUMentum Pregnancy Program intervention group [52]. Virtually all pregnant women have regular contact with health care professionals, regardless of their psychosocial well-being, and that may also have a positive effect on any depressive symptoms. This is consistent with findings of a recent individual network meta-analysis that the effectiveness of guided iCBT interventions were higher for individuals with moderate to severe depression, but unguided iCBT showed similar effectiveness for individuals with mild or subthreshold depression [72].

### Limitations

Although this review was conducted with high scientific rigor and we carried out a full quality appraisal of the included studies, there are several limitations that need to be taken into account when interpreting the findings. We only included papers published in English, and this may have meant that some potential studies in other languages or grey literature from other databases were not included. All except one of the included studies had a high risk of bias. They also used participant-reported measures for most of the outcomes, which can be influenced by knowledge of the intervention being received. Using the ROB2, this resulted in at least some concern of a risk of bias for all the studies. As only 1 study reported an infant-related outcome, namely infant temperament, and only 1 study assessed mother-infant attachment, meaningful discussions about infant outcomes and maternal attachment are not possible. In addition, our review only focused on CBT and did not include interpersonal psychotherapy, although both psychotherapies were included in the US Preventive Services Task Force treatment guideline for perinatal depression that was issued in 2019 [11]. A recent review found that interpersonal psychotherapy interventions for psychological distress in perinatal populations were limited to those delivered face-to-face or by telephone [85]. The nonstandardized description of interventions in our review also made it difficult to compare them. This calls for more standardized descriptions of interventions, such as using the Template for Intervention Description and Replication checklist [86]. Future studies could also explore various acceptability outcomes before, during, and after interventions are delivered. This could be done by using the 7 components of the Theoretical Framework of Acceptability: affective attitude, burden, perceived effectiveness, ethicality, intervention coherence, opportunity costs, and self-efficacy [46].

### Conclusion

This systematic review suggests promising evidence for the potential efficacy, acceptability, and integration of digitalized CBT interventions that start during pregnancy. However, all except one of the studies included in this review recorded a high overall risk of bias. In future, we need high-quality studies with larger population-based samples to comprehensively analyze the efficacy of the interventions and explore the mechanisms of change. Digital interventions may have significant global implications when planning effective, nonstigmatizing, and cost-effective mental health treatment to prevent the long-term consequences of psychosocial problems in pregnancy. These could also have other wide-ranging clinical applications. Furthermore, there is an urgent need to study digital and remote interventions, as mental health services struggle to deal with the considerable increase in demand due to the COVID-19 pandemic. 

## Appendix

Multimedia Appendix 1 Search strings and databases.

Multimedia Appendix 2 List of excluded studies after full-text review.

Multimedia Appendix 3 Attempts to contact authors.

## Abbreviations

CBT: cognitive behavioral therapy
COM-B: Capability, Opportunity, Motivation, Behavior
EPDS: Edinburgh Postnatal Depression Scale
iCBT: internet-based CBT
MADRS: Montgomery-Åsberg Depression Rating Scale
PHQ: Patient Health Questionnaire
PICOS: population, intervention, comparator, outcome, and study design
PRISMA: Preferred Reporting Items of Systematic Reviews and Meta-analyses
RCT: randomized controlled trial
ROB2: Revised Cochrane risk-of-bias tool for randomized trials

## References

[1] Yin X, Sun N, Jiang N, Xu X, Gan Y, Zhang J, Qiu L, Yang C, Shi X, Chang J, Gong Y (2021). Prevalence and associated factors of antenatal depression: Systematic reviews and meta-analyses. Clin Psychol Rev.

[2] Wilcox M, McGee B, Ionescu D, Leonte M, LaCross L, Reps J, Wildenhaus K (2021). Perinatal depressive symptoms often start in the prenatal rather than postpartum period: results from a longitudinal study. Arch Womens Ment Health.

[3] Faisal-Cury A, Menezes P (2012). Antenatal depression strongly predicts postnatal depression in primary health care. Braz J Psychiatry.

[4] Fekadu Dadi A, Miller ER, Mwanri L (2020). Antenatal depression and its association with adverse birth outcomes in low and middle-income countries: A systematic review and meta-analysis. PLoS One.

[5] Yedid Sion M, Harlev A, Weintraub AY, Sergienko R, Sheiner E (2016). Is antenatal depression associated with adverse obstetric and perinatal outcomes?. J Matern Fetal Neonatal Med.

[6] Stein A, Pearson R, Goodman S, Rapa E, Rahman A, McCallum M, Howard LM, Pariante CM (2014). Effects of perinatal mental disorders on the fetus and child. The Lancet.

[7] Hay Rebecca E, Reynolds Jess E, Grohs Melody N, Paniukov Dmitrii, Giesbrecht Gerald F, Letourneau Nicole, Dewey Deborah, Lebel Catherine (2020). Amygdala-prefrontal structural connectivity mediates the relationship between prenatal depression and behavior in preschool boys. J Neurosci.

[8] Posner J, Cha J, Roy AK, Peterson BS, Bansal R, Gustafsson HC, Raffanello E, Gingrich J, Monk C (2016). Alterations in amygdala-prefrontal circuits in infants exposed to prenatal maternal depression. Transl Psychiatry.

[9] Amiel Castro RT, Pinard Anderman C, Glover V, O'Connor TG, Ehlert U, Kammerer M (2017). Associated symptoms of depression: patterns of change during pregnancy. Arch Womens Ment Health.

[10] Weissman MM (2020). Intergenerational study of depression: a convergence of findings and opportunities. Psychol Med.

[11] Curry SJ, Krist AH, Owens DK, Barry MJ, Caughey AB, Davidson KW, Doubeni CA, Epling JW, Grossman DC, Kemper AR, Kubik M, Landefeld CS, Mangione CM, Silverstein M, Simon MA, Tseng C, Wong JB, US Preventive Services Task Force (2019). Interventions to prevent perinatal depression: US Preventive Services Task Force Recommendation Statement. JAMA.

[12] Le H, Perry DF, Stuart EA (2011). Randomized controlled trial of a preventive intervention for perinatal depression in high-risk Latinas. J Consult Clin Psychol.

[13] Gingrich J, Malm H, Ansorge M, Brown A, Sourander A, Suri D, Teixeira CM, Caffrey Cagliostro MK, Mahadevia D, Weissman MM (2017). New insights into how serotonin selective reuptake inhibitors shape the developing brain. Birth Defects Res.

[14] Malm H, Brown AS, Gissler M, Gyllenberg D, Hinkka-Yli-Salomäki S, McKeague IW, Weissman M, Wickramaratne P, Artama M, Gingrich JA, Sourander A (2016). Gestational exposure to selective serotonin reuptake inhibitors and offspring psychiatric disorders: a national register-based study. J Am Acad Child Adolesc Psychiatry.

[15] Brown AS, Gyllenberg D, Malm H, McKeague IW, Hinkka-Yli-Salomäki S, Artama M, Gissler M, Cheslack-Postava K, Weissman MM, Gingrich JA, Sourander A (2016). Association of selective serotonin reuptake inhibitor exposure during pregnancy with speech, scholastic, and motor disorders in offspring. JAMA Psychiatry.

[16] Da Costa D, Zelkowitz P, Nguyen TV, Deville-Stoetzel JB (2018). Mental health help-seeking patterns and perceived barriers for care among nulliparous pregnant women. Arch Womens Ment Health.

[17] Goodman J (2009). Women's attitudes, preferences, and perceived barriers to treatment for perinatal depression. Birth.

[18] Jones A (2019). Help seeking in the perinatal period: a review of barriers and facilitators. Soc Work Public Health.

[19] Kopelman RC, Moel J, Mertens C, Stuart S, Arndt S, O'Hara MW (2008). Barriers to care for antenatal depression. Psychiatr Serv.

[20] Singla DR, Lawson A, Kohrt BA, Jung JW, Meng Z, Ratjen C, Zahedi N, Dennis C, Patel V (2021). Implementation and effectiveness of nonspecialist-delivered interventions for perinatal mental health in high-income countries: a systematic review and meta-analysis. JAMA Psychiatry.

[21] World Health Organization (2018). Mental Health Atlas 2017. https://www.who.int/publications/i/item/9789241514019.

[22] Haggerty R, Mrazek P, Institute of Medicine (US) Committee on Prevention of Mental Disorders (1994). Reducing risks for mental disorders: Frontiers for preventive intervention research.

[23] San Martin Porter MA, Kisely S, Alati R (2019). Screening for perinatal depression and predictors of underscreening: findings of the Born in Queensland study. Med J Aust.

[24] Kazdin AE, Blase SL (2011). Rebooting psychotherapy research and practice to reduce the burden of mental illness. Perspect Psychol Sci.

[25] Kazdin AE, Rabbitt SM (2013). Novel models for delivering mental health services and reducing the burdens of mental illness. Clinical Psychological Science.

[26] Ristkari T, Kurki M, Suominen A, Gilbert S, Sinokki A, Kinnunen M, Huttunen J, McGrath P, Sourander A (2019). Web-based parent training intervention with telephone coaching for disruptive behavior in 4-year-old children in real-world practice: implementation study. J Med Internet Res.

[27] Sourander A, McGrath PJ, Ristkari T, Cunningham C, Huttunen J, Lingley-Pottie P, Hinkka-Yli-Salomäki S, Kinnunen M, Vuorio J, Sinokki A, Fossum S, Unruh A (2016). Internet-assisted parent training intervention for disruptive behavior in 4-year-old children: a randomized clinical trial. JAMA Psychiatry.

[28] Sourander A, McGrath P, Ristkari T, Cunningham C, Huttunen J, Hinkka-Yli-Salomäki S, Kurki M, Lingley-Pottie P (2018). Two-year follow-Up of internet and telephone assisted parent training for disruptive behavior at age 4. J Am Acad Child Adolesc Psychiatry.

[29] Gülmezoglu AM, Ammerdorffer A, Narasimhan M, Wilson AN, Vogel JP, Say L, Tunçalp Ö (2020). Self-care and remote care during pregnancy: a new paradigm?. Health Res Policy Syst.

[30] Li C, Sun X, Li Q, Sun Q, Wu B, Duan D (2020). Role of psychotherapy on antenatal depression, anxiety, and maternal quality of life: A meta-analysis. Medicine (Baltimore).

[31] Loughnan S, Joubert A, Grierson A, Andrews G, Newby J (2019). Internet-delivered psychological interventions for clinical anxiety and depression in perinatal women: a systematic review and meta-analysis. Arch Womens Ment Health.

[32] Lee E, Denison F, Hor K, Reynolds R (2016). Web-based interventions for prevention and treatment of perinatal mood disorders: a systematic review. BMC Pregnancy Childbirth.

[33] Nillni Y, Mehralizade A, Mayer L, Milanovic S (2018). Treatment of depression, anxiety, and trauma-related disorders during the perinatal period: A systematic review. Clin Psychol Rev.

[34] Smoktunowicz E, Barak A, Andersson G, Banos R, Berger T, Botella C, Dear BF, Donker T, Ebert DD, Hadjistavropoulos H, Hodgins DC, Kaldo V, Mohr DC, Nordgreen T, Powers MB, Riper H, Ritterband LM, Rozental A, Schueller SM, Titov N, Weise C, Carlbring P (2020). Consensus statement on the problem of terminology in psychological interventions using the internet or digital components. Internet Interv.

[35] Moher D, Liberati A, Tetzlaff J, Altman D, PRISMA Group (2009). Preferred reporting items for systematic reviews and meta-analyses: the PRISMA statement. PLoS Med.

[36] Campbell M, McKenzie J, Sowden A, Katikireddi S, Brennan S, Ellis S, Hartmann-Boyce J, Ryan R, Shepperd S, Thomas J, Welch V, Thomson H (2020). Synthesis without meta-analysis (SWiM) in systematic reviews: reporting guideline. BMJ.

[37] Wohlin C (2014). Guidelines for snowballing in systematic literature studies and a replication in software engineering. https://dl.acm.org/doi/10.1145/2601248.2601268.

[38] Amir-Behghadami M, Janati A (2020). Population, Intervention, Comparison, Outcomes and Study (PICOS) design as a framework to formulate eligibility criteria in systematic reviews. Emerg Med J.

[39] Luik AI, van der Zweerde T, van Straten A, Lancee J (2019). Digital delivery of cognitive behavioral therapy for insomnia. Curr Psychiatry Rep.

[40] Wolters LH, Op de Beek V, Weidle B, Skokauskas N (2017). How can technology enhance cognitive behavioral therapy: the case of pediatric obsessive compulsive disorder. BMC Psychiatry.

[41] Lilienfeld S, McKay D, Hollon S (2018). Why randomised controlled trials of psychological treatments are still essential. Lancet Psychiatry.

[42] Bristow D, Carter L, Martin S (2015). Using evidence to improve policy and practice: the UK What Works Centres. Contemporary Social Science.

[43] Sterne J, Savović J, Page M, Elbers R, Blencowe N, Boutron I, Cates CJ, Cheng HY, Corbett MS, Eldridge SM, Emberson JR, Hernán MA, Hopewell S, Hróbjartsson A, Junqueira DR, Jüni P, Kirkham JJ, Lasserson T, Li T, McAleenan A, Reeves BC, Shepperd S, Shrier I, Stewart LA, Tilling K, White IR, Whiting PF, Higgins JPT (2019). RoB 2: a revised tool for assessing risk of bias in randomised trials. BMJ.

[44] Effect Size Calculator. Cambridge University Press & Assessment Center for Evaluation & Monitoring.

[45] Lipsey MW, Wilson DB (1993). The efficacy of psychological, educational, and behavioral treatment: Confirmation from meta-analysis. American Psychologist.

[46] Sekhon M, Cartwright M, Francis J (2017). Acceptability of healthcare interventions: an overview of reviews and development of a theoretical framework. BMC Health Serv Res.

[47] Barrera A, Wickham R, Muñoz RF (2015). Online prevention of postpartum depression for Spanish- and English-speaking pregnant women: A pilot randomized controlled trial. Internet Interv.

[48] Duffecy J, Grekin R, Hinkel H, Gallivan N, Nelson G, O'Hara M (2019). A group-based online intervention to prevent postpartum depression (Sunnyside): feasibility randomized controlled trial. JMIR Ment Health.

[49] Forsell E, Bendix M, Holländare F, Szymanska von Schultz B, Nasiell J, Blomdahl-Wetterholm M, Eriksson C, Kvarned S, Lindau van der Linden J, Söderberg E, Jokinen J, Wide K, Kaldo V (2017). Internet delivered cognitive behavior therapy for antenatal depression: A randomised controlled trial. J Affect Disord.

[50] Guo L, Zhang J, Mu L, Ye Z (2020). Preventing postpartum depression with mindful self-compassion intervention: a randomized control study. J Nerv Ment Dis.

[51] Haga S, Drozd F, Lisøy Carina, Wentzel-Larsen T, Slinning K (2019). Mamma Mia - A randomized controlled trial of an internet-based intervention for perinatal depression. Psychol Med.

[52] Loughnan S, Sie A, Hobbs M, Joubert A, Smith J, Haskelberg H, Mahoney AEJ, Kladnitski N, Holt CJ, Milgrom J, Austin MP, Andrews G, Newby JM (2019). A randomized controlled trial of 'MUMentum Pregnancy': Internet-delivered cognitive behavioral therapy program for antenatal anxiety and depression. J Affect Disord.

[53] Sun Y, Li Y, Wang J, Chen Q, Bazzano AN, Cao F (2021). Effectiveness of smartphone-based mindfulness training on maternal perinatal depression: randomized controlled trial. J Med Internet Res.

[54] Montgomery SA, Asberg M (1979). A new depression scale designed to be sensitive to change. Br J Psychiatry.

[55] Cox JL, Holden JM, Sagovsky R (1987). Detection of postnatal depression. Development of the 10-item Edinburgh Postnatal Depression Scale. Br J Psychiatry.

[56] Kroenke K, Spitzer RL (2002). The PHQ-9: a new depression diagnostic and severity measure. Psychiatric Annals.

[57] Radloff LS (2016). The CES-D Scale. Applied Psychological Measurement.

[58] Beck AT, Steer RA, Brown GK (1996). Manual for the Beck Depression Inventory-II.

[59] Putnam SP, Helbig AL, Gartstein MA, Rothbart MK, Leerkes E (2014). Development and assessment of short and very short forms of the infant behavior questionnaire-revised. J Pers Assess.

[60] Condon J (1993). The assessment of antenatal emotional attachment: development of a questionnaire instrument. Br J Med Psychol.

[61] Spitzer RL, Kroenke K, Williams JBW, Löwe B (2006). A brief measure for assessing generalized anxiety disorder: the GAD-7. Arch Intern Med.

[62] Spielberger CD, Gorsuch RL, Lushene R, Vagg PR, Jacobs GA (1983). Manual for the State-Trait Anxiety Inventory.

[63] Watson D, O'Hara MW, Simms LJ, Kotov R, Chmielewski M, McDade-Montez EA, Gamez W, Stuart S (2007). Development and validation of the Inventory of Depression and Anxiety Symptoms (IDAS). Psychol Assess.

[64] Drozd F, Haga S, Brendryen H, Slinning K (2015). An internet-based intervention (Mamma Mia) for postpartum depression: mapping the development from theory to practice. JMIR Res Protoc.

[65] Haga S, Drozd F, Brendryen H, Slinning K (2013). Mamma mia: a feasibility study of a web-based intervention to reduce the risk of postpartum depression and enhance subjective well-being. JMIR Res Protoc.

[66] Andersson G (2018). Internet interventions: Past, present and future. Internet Interv.

[67] Webb C, Rosso I, Rauch S (2017). Internet-based cognitive-behavioral therapy for depression: current progress and future directions. Harv Rev Psychiatry.

[68] Ellis L, Roberts L (2020). Exploring the use and quality of Internet discussion forums in pregnancy: A qualitative analysis. Birth.

[69] Richards D, Richardson T (2012). Computer-based psychological treatments for depression: a systematic review and meta-analysis. Clin Psychol Rev.

[70] Andersson G, Cuijpers P (2009). Internet-based and other computerized psychological treatments for adult depression: a meta-analysis. Cogn Behav Ther.

[71] Spek V, Cuijpers P, Nyklícek Ivan, Riper H, Keyzer J, Pop V (2007). Internet-based cognitive behaviour therapy for symptoms of depression and anxiety: a meta-analysis. Psychol Med.

[72] Karyotaki E, Efthimiou O, Miguel C, Bermpohl FMG, Furukawa T, Cuijpers P, Individual Patient Data Meta-Analyses for Depression (IPDMA-DE) Collaboration (2021). Internet-based cognitive behavioral therapy for depression: a systematic review and individual patient data network meta-analysis. JAMA Psychiatry.

[73] Savory K, Garay S, Sumption L, Kelleher J, Daughters K, Janssen A, Van Goozen S, John RM (2020). Prenatal symptoms of anxiety and depression associated with sex differences in both maternal perceptions of one year old infant temperament and researcher observed infant characteristics. J Affect Disord.

[74] Lahti M, Savolainen K, Tuovinen S, Pesonen A, Lahti J, Heinonen K, Hämäläinen E, Laivuori H, Villa PM, Reynolds RM, Kajantie E, Räikkönen K (2017). Maternal Depressive Symptoms During and After Pregnancy and Psychiatric Problems in Children. J Am Acad Child Adolesc Psychiatry.

[75] Räikkönen K, Pesonen A, O'Reilly Jr, Tuovinen S, Lahti M, Kajantie E, Villa P, Laivuori H, Hämäläinen E, Seckl JR, Reynolds RM (2015). Maternal depressive symptoms during pregnancy, placental expression of genes regulating glucocorticoid and serotonin function and infant regulatory behaviors. Psychol Med.

[76] McGrath J, Records K, Rice M (2008). Maternal depression and infant temperament characteristics. Infant Behav Dev.

[77] Talge N, Neal C, Glover V, Early Stress‚ Translational Research and Prevention Science Network: Fetal and Neonatal Experience on Child and Adolescent Mental Health (2007). Antenatal maternal stress and long-term effects on child neurodevelopment: how and why?. J Child Psychol Psychiatry.

[78] Davis E, Glynn L, Schetter C, Hobel C, Chicz-Demet A, Sandman C (2007). Prenatal exposure to maternal depression and cortisol influences infant temperament. J Am Acad Child Adolesc Psychiatry.

[79] Van den Bergh BRH, Mulder E, Mennes M, Glover V (2005). Antenatal maternal anxiety and stress and the neurobehavioural development of the fetus and child: links and possible mechanisms. A review. Neurosci Biobehav Rev.

[80] Austin M (2004). Antenatal screening and early intervention for "perinatal" distress, depression and anxiety: where to from here?. Arch Womens Ment Health.

[81] Michie S, van Stralen MM, West R (2011). The behaviour change wheel: a new method for characterising and designing behaviour change interventions. Implement Sci.

[82] Phelan S (2010). Pregnancy: a "teachable moment" for weight control and obesity prevention. Am J Obstet Gynecol.

[83] Olander E, Darwin Z, Atkinson L, Smith D, Gardner B (2016). Beyond the 'teachable moment' - A conceptual analysis of women's perinatal behaviour change. Women Birth.

[84] Olander EK, Smith DM, Darwin Z (2018). Health behaviour and pregnancy: a time for change. J Reprod Infant Psychol.

[85] Bright KS, Charrois EM, Mughal MK, Wajid A, McNeil D, Stuart S, Hayden KA, Kingston D (2020). Interpersonal psychotherapy to reduce psychological distress in perinatal women: a systematic review. Int J Environ Res Public Health.

[86] Hoffmann T, Glasziou P, Boutron I, Milne R, Perera R, Moher D, Altman DG, Barbour V, Macdonald H, Johnston M, Lamb SE, Dixon-Woods M, McCulloch P, Wyatt JC, Chan AW, Michie S (2014). Better reporting of interventions: template for intervention description and replication (TIDieR) checklist and guide. BMJ.

