# Supplementary material for: Digitalized Cognitive Behavioral Interventions for Depressive Symptoms During Pregnancy: Systematic Review
Source: J Med Internet Res. 2022 Feb 23;24(2):e33337. doi: 10.2196/33337 (PMC8908191; doi:10.2196/33337)
Supplement: Multimedia Appendix 1 [file jmir_v24i2e33337_app1.docx]

**Supplementary File 1: Search strings and databases**

**1. Web of Science**

(TI=(Depress*) OR AB=(Depress*)) AND (TI=(pregnan* OR perinatal OR peripartum OR prenatal OR antenatal OR antepartum OR postpartum OR postnatal OR maternal OR birth) OR AB=(pregnan* OR perinatal OR peripartum OR prenatal OR antenatal OR antepartum OR postpartum OR postnatal OR maternal OR birth)) AND (TI=("cognitive behavio*" OR CBT OR iCBT OR cCBT OR dCBT OR tCBT OR internet* OR computer* OR online* OR web* OR digital* OR mobile* OR virtual* OR "augment* reality" OR "conversational agent" OR chatbot* OR app OR video* OR tele* OR technolog* OR e-therapy OR e-mental OR e-health OR cyber OR cyberpsychology OR cybertherapy) OR AB=("cognitive behavio*" OR CBT OR iCBT OR cCBT OR dCBT OR tCBT OR internet* OR computer* OR online* OR web* OR digital* OR mobile* OR virtual* OR "augment* reality" OR "conversational agent" OR chatbot* OR app OR video* OR tele* OR technolog* OR e-therapy OR e-mental OR e-health OR cyber OR cyberpsychology OR cybertherapy)) AND (TI=("randomi* control*" OR RCT OR randomized OR randomised OR randomly OR “double blind” OR “single blind”) OR AB=("randomi* control*" OR RCT OR randomized OR randomised OR randomly OR “double blind” OR “single blind”))

**2. Cochrane Central Register of Controlled Trials**

Depress* AND (pregnan* OR perinatal OR peripartum OR prenatal OR antenatal OR antepartum OR postpartum OR postnatal OR maternal OR birth) AND ("cognitive behavio*" OR CBT OR iCBT OR cCBT OR dCBT OR tCBT OR internet* OR computer* OR online* OR web* OR digital* OR mobile* OR virtual* OR "augment* reality" OR "conversational agent" OR chatbot* OR app OR video* OR tele* OR technolog* OR e-therapy OR e-mental OR e-health OR cyber OR cyberpsychology OR cybertherapy) AND ("randomi* NEXT control*" OR RCT OR randomized OR randomised OR randomly OR “double blind” OR “single blind”)

**3. EBSCOhost (CINAHL)**

(TI depress* OR AB depress*) AND (TI pregnan* OR TI perinatal OR TI peripartum OR TI prenatal OR TI antenatal OR TI antepartum OR TI postpartum OR TI postnatal OR TI maternal OR TI birth OR AB pregnan* OR AB perinatal OR AB peripartum OR AB prenatal OR AB antenatal OR AB antepartum OR AB postpartum OR AB postnatal OR AB maternal OR AB birth) AND (TI 'cognitive behavio*' OR TI cbt OR TI icbt OR TI ccbt OR TI dcbt OR TI tcbt OR TI internet* OR TI computer* OR TI online* OR TI web* OR TI digital* OR TI mobile* OR TI virtual* OR TI 'augment* reality' OR TI 'conversational agent' OR TI chatbot* OR TI app OR TI video* OR TI tele* OR TI technolog* OR TI 'e therapy' OR TI 'e mental' OR TI 'e health' OR TI cyber OR TI cyberpsychology OR TI cybertherapy OR AB 'cognitive behavio*' OR AB cbt OR AB icbt OR AB ccbt OR AB dcbt OR AB tcbt OR AB internet* OR AB computer* OR AB online* OR AB web* OR AB digital* OR AB mobile* OR AB virtual* OR AB 'augment* reality' OR AB 'conversational agent' OR AB chatbot* OR AB app OR AB video* OR AB tele* OR AB technolog* OR AB 'e therapy' OR AB 'e mental' OR AB 'e health' OR AB cyber OR AB cyberpsychology OR AB cybertherapy) AND (TI 'randomi* control*' OR TI rct OR TI randomized OR TI randomised OR TI randomly OR TI “double blind” OR TI “single blind” OR AB 'randomi* control*' OR AB rct OR AB randomized OR AB randomised OR AB randomly OR AB “double blind” OR AB “single blind”)

**4. EBSCOhost (MEDLINE)**

(TI depress* OR AB depress*) AND (TI pregnan* OR TI perinatal OR TI peripartum OR TI prenatal OR TI antenatal OR TI antepartum OR TI postpartum OR TI postnatal OR TI maternal OR TI birth OR AB pregnan* OR AB perinatal OR AB peripartum OR AB prenatal OR AB antenatal OR AB antepartum OR AB postpartum OR AB postnatal OR AB maternal OR AB birth) AND (TI 'cognitive behavio*' OR TI cbt OR TI icbt OR TI ccbt OR TI dcbt OR TI tcbt OR TI internet* OR TI computer* OR TI online* OR TI web* OR TI digital* OR TI mobile* OR TI virtual* OR TI 'augment* reality' OR TI 'conversational agent' OR TI chatbot* OR TI app OR TI video* OR TI tele* OR TI technolog* OR TI 'e therapy' OR TI 'e mental' OR TI 'e health' OR TI cyber OR TI cyberpsychology OR TI cybertherapy OR AB 'cognitive behavio*' OR AB cbt OR AB icbt OR AB ccbt OR AB dcbt OR AB tcbt OR AB internet* OR AB computer* OR AB online* OR AB web* OR AB digital* OR AB mobile* OR AB virtual* OR AB 'augment* reality' OR AB 'conversational agent' OR AB chatbot* OR AB app OR AB video* OR AB tele* OR AB technolog* OR AB 'e therapy' OR AB 'e mental' OR AB 'e health' OR AB cyber OR AB cyberpsychology OR AB cybertherapy) AND (TI 'randomi* control*' OR TI rct OR TI randomized OR TI randomised OR TI randomly OR TI “double blind” OR TI “single blind” OR AB 'randomi* control*' OR AB rct OR AB randomized OR AB randomised OR AB randomly OR AB “double blind” OR AB “single blind”)

**5. Embase**

depress*:ti,ab AND (pregnan*:ti,ab OR perinatal:ti,ab OR peripartum:ti,ab OR prenatal:ti,ab OR antenatal:ti,ab OR antepartum:ti,ab OR postpartum:ti,ab OR postnatal:ti,ab OR maternal:ti,ab OR birth:ti,ab) AND ('cognitive behavio*':ti,ab OR cbt:ti,ab OR icbt:ti,ab OR ccbt:ti,ab OR dcbt:ti,ab OR tcbt:ti,ab OR internet*:ti,ab OR computer*:ti,ab OR online*:ti,ab OR web*:ti,ab OR digital*:ti,ab OR mobile*:ti,ab OR virtual*:ti,ab OR 'augment* reality':ti,ab OR 'conversational agent':ti,ab OR chatbot*:ti,ab OR app:ti,ab OR video*:ti,ab OR tele*:ti,ab OR technolog*:ti,ab OR 'e therapy':ti,ab OR 'e mental':ti,ab OR 'e health':ti,ab OR cyber:ti,ab OR cyberpsychology:ti,ab OR 'cybertherapy':ti,ab OR cybertherapy:ti,ab) AND ('randomi* control*':ti,ab OR rct:ti,ab OR randomized:ti,ab OR randomised:ti,ab OR randomly:ti,ab OR 'double blind':ti,ab OR 'single blind':ti,ab)

**6. EBSCOhost (APA PsychInfo)**

(TI depress* OR AB depress*) AND (TI pregnan* OR TI perinatal OR TI peripartum OR TI prenatal OR TI antenatal OR TI antepartum OR TI postpartum OR TI postnatal OR TI maternal OR TI birth OR AB pregnan* OR AB perinatal OR AB peripartum OR AB prenatal OR AB antenatal OR AB antepartum OR AB postpartum OR AB postnatal OR AB maternal OR AB birth) AND (TI 'cognitive behavio*' OR TI cbt OR TI icbt OR TI ccbt OR TI dcbt OR TI tcbt OR TI internet* OR TI computer* OR TI online* OR TI web* OR TI digital* OR TI mobile* OR TI virtual* OR TI 'augment* reality' OR TI 'conversational agent' OR TI chatbot* OR TI app OR TI video* OR TI tele* OR TI technolog* OR TI 'e therapy' OR TI 'e mental' OR TI 'e health' OR TI cyber OR TI cyberpsychology OR TI cybertherapy OR AB 'cognitive behavio*' OR AB cbt OR AB icbt OR AB ccbt OR AB dcbt OR AB tcbt OR AB internet* OR AB computer* OR AB online* OR AB web* OR AB digital* OR AB mobile* OR AB virtual* OR AB 'augment* reality' OR AB 'conversational agent' OR AB chatbot* OR AB app OR AB video* OR AB tele* OR AB technolog* OR AB 'e therapy' OR AB 'e mental' OR AB 'e health' OR AB cyber OR AB cyberpsychology OR AB cybertherapy) AND (TI 'randomi* control*' OR TI rct OR TI randomized OR TI randomised OR TI randomly OR TI “double blind” OR TI “single blind” OR AB 'randomi* control*' OR AB rct OR AB randomized OR AB randomised OR AB randomly OR AB “double blind” OR AB “single blind”)

**7. Scopus**

TITLE-ABS ( depress* ) AND TITLE-ABS ( pregnan* OR perinatal OR peripartum OR prenatal OR antenatal OR antepartum OR postpartum OR postnatal OR maternal OR birth ) AND TITLE-ABS ( "cognitive behavio*" OR cbt OR icbt OR ccbt OR dcbt OR tcbt OR internet* OR computer* OR online* OR web* OR digital* OR mobile* OR virtual* OR "augment* reality" OR "conversational agent" OR chatbot* OR app OR video* OR tele* OR technolog* OR e-therapy OR e-mental OR e-health OR cyber OR cyberpsychology OR cybertherapy ) AND TITLE-ABS ( "randomi* control*" OR rct OR randomized OR randomised OR randomly OR “double blind” OR “single blind”)

**8. ClinicalTrials.gov**

Condition or disease Maternal depression OR perinatal depression OR peripartum depression OR prenatal depression OR antenatal depression OR antepartum depression OR postpartum depression OR postnatal depression

Applied Filters Study type: Interventional studies (Clinical trials)

Age Group: Adult (18-64)

Intervention/treatment: Behavioral

Completed Trials

**9. EBSCO Open Dissertations**

(TI depress* OR AB depress*) AND (TI pregnan* OR TI perinatal OR TI peripartum OR TI prenatal OR TI antenatal OR TI antepartum OR TI postpartum OR TI postnatal OR TI maternal OR TI birth OR AB pregnan* OR AB perinatal OR AB peripartum OR AB prenatal OR AB antenatal OR AB antepartum OR AB postpartum OR AB postnatal OR AB maternal OR AB birth) AND (TI 'cognitive behavio*' OR TI cbt OR TI icbt OR TI ccbt OR TI dcbt OR TI tcbt OR TI internet* OR TI computer* OR TI online* OR TI web* OR TI digital* OR TI mobile* OR TI virtual* OR TI 'augment* reality' OR TI 'conversational agent' OR TI chatbot* OR TI app OR TI video* OR TI tele* OR TI technolog* OR TI 'e therapy' OR TI 'e mental' OR TI 'e health' OR TI cyber OR TI cyberpsychology OR TI cybertherapy OR AB 'cognitive behavio*' OR AB cbt OR AB icbt OR AB ccbt OR AB dcbt OR AB tcbt OR AB internet* OR AB computer* OR AB online* OR AB web* OR AB digital* OR AB mobile* OR AB virtual* OR AB 'augment* reality' OR AB 'conversational agent' OR AB chatbot* OR AB app OR AB video* OR AB tele* OR AB technolog* OR AB 'e therapy' OR AB 'e mental' OR AB 'e health' OR AB cyber OR AB cyberpsychology OR AB cybertherapy) AND (TI 'randomi* control*' OR TI rct OR TI randomized OR TI randomised OR TI randomly OR TI “double blind” OR TI “single blind” OR AB 'randomi* control*' OR AB rct OR AB randomized OR AB randomised OR AB randomly OR AB “double blind” OR AB “single blind”)
