# Supplementary material for: Digitalized Cognitive Behavioral Interventions for Depressive Symptoms During Pregnancy: Systematic Review
Source: J Med Internet Res. 2022 Feb 23;24(2):e33337. doi: 10.2196/33337 (PMC8908191; doi:10.2196/33337)
Supplement: Multimedia Appendix 2 [file jmir_v24i2e33337_app2.docx]

**Supplementary File 2: List of excluded studies after full-text review**

1. Include partners/adolecents [1-3]
2. Not CBT [4-14]
3. Not digitalized [15-40]
4. Intervention begins postnatally [41-65]
5. Depression not primary outcome [66-78]
6. Not RCT/other designs [79-99]
7. No/unable to obtain fulltext [100-102]
8. Other publication of same intervention [103, 104]

1. Chyzzy B, Dennis C-L. 16. Mobile Phone-Based Peer Support In The Prevention of Postpartum Depression Among Adolescent Mothers: A Pilot Randomized Controlled Trial...SAHM Annual Meeting, Psychological Well-Being: International Transcultural Perspectives, March 6-9, 2019, Washin. Journal of Adolescent Health. 2019;64:S8-S9. PMID: 134422981. Language: English. Entry Date: 20190205. Revision Date: 20190710. Publication Type: Article. doi: 10.1016/j.jadohealth.2018.10.030.

2. Collado MAO, Saez M, Favrod J, Hatem M. Antenatal psychosomatic programming to reduce postpartum depression risk and improve childbirth outcomes: a randomized controlled trial in Spain and France. BMC Pregnancy and Childbirth. 2014;14:12-. doi: 10.1186/1471-2393-14-22.

3. Scazufca M, Curi AF, Mogadouro M, Ramirez A, Fenerich S, Almeida S, et al. Increasing the likelihood of identification and treatment of depression during pregnancy as a strategy to prevent mental health problems during childhood and adolescence. European Child and Adolescent Psychiatry. 2013;22(2):S113-S4. doi: 10.1007/s00787-013-0423-9.

4. Bullock L, Everett KD, Mullen PD, Geden E, Longo DR, Madsen R. Baby BEEP: A randomized controlled trial of nurses' individualized social support for poor rural pregnant smokers. Maternal and child health journal. 2009;13(3):395-406. doi: 10.1007/s10995-008-0363-z.

5. Chan KL, Leung WC, Tiwari A, Or KL, Ip P. Using Smartphone-Based Psychoeducation to Reduce Postnatal Depression Among First-Time Mothers: Randomized Controlled Trial. JMIR mHealth and uHealth. 2019;7(5):11-. doi: 10.2196/12794.

6. Chaudhry I, Chaudhry N, Khalid T, Kiran T, Husain M, Furber C, et al. Development and assessment of smartphone based intervention (TechMotherCare) for maternal depression in Pakistan. Journal of Psychosomatic Research. 2020;133. doi: 10.1016/j.jpsychores.2020.110029.

7. Dennis CL, Ravitz P, Grigoriadis S, Jovellanos M, Hodnett E, Ross L, et al. A multi-site randomized controlled trial to evaluate the effect of telephone-based interpersonal psychotherapy by trained nurses for the treatment of postpartum depression. Archives of Women's Mental Health. 2015;18(2):288-9. doi: 10.1007/s00737-014-0488-6.

8. Donovan V, Cope C, Lyons A, Thompson PJ, Martin B, Whittle MJ, et al. Effects of different media to supplement a prenatal specialist consultation: A single-centre randomised controlled trial. Journal of Obstetrics and Gynaecology. 2003;23(sup1):S12-S. doi: 10.1080/718591789.

9. Gao L, Chan SW, Li X, Chen S, Hao Y. Evaluation of an interpersonal-psychotherapy-oriented childbirth education programme for Chinese first-time childbearing women: A randomised controlled trial. International journal of nursing studies. 2010;47(10):1208-16. doi: 10.1016/j.ijnurstu.2010.03.002.

10. Hall H, Munk N, Carr B, Fogarty S, Cant R, Holton S, et al. Maternal mental health and partner-delivered massage: A pilot study. Women and Birth. 2020. doi: 10.1016/j.wombi.2020.05.003.

11. Leung SSK, Lam TH. Group antenatal intervention to reduce perinatal stress and depressive symptoms related to intergenerational conflicts: A randomized controlled trial. International journal of nursing studies. 2012;49(11):1391-402. doi: 10.1016/j.ijnurstu.2012.06.014.

12. Matthey S, Kavanagh DJ, Howie P, Barnett B, Charles M. Prevention of postnatal distress or depression: An evaluation of an intervention at preparation for parenthood classes. Journal of Affective Disorders. 2004;79(1-3):113-26. doi: 10.1016/S0165-0327(02)00362-2.

13. Shorey S, Chee CYI, Ng ED, Lau Y, Dennis C-L, Chan YH. Evaluation of a technology-based peer-support intervention program for preventing postnatal depression (Part 1): Randomized controlled trial. Journal of Medical Internet Research. 2019;21(8). PMID: 2019-55464-001.

14. Huckins JF, da Silva AW, Wang W, Hedlund E, Rogers C, Nepal SK, et al. Mental health and behavior of college students during the early phases of the COVID-19 pandemic: Longitudinal smartphone and ecological momentary assessment study. Journal of Medical Internet Research. 2020;22(6). doi: 10.2196/20185.

15. Austin MP, Frilingos M, Lumley J, Hadzi-Pavlovic D, Roncolato W, Acland S, et al. Brief antenatal cognitive behaviour therapy group intervention for the-prevention of postnatal depression and anxiety: A randomised controlled trial. Journal of Affective Disorders. 2008;105(1-3):35-44. doi: 10.1016/j.jad.2007.04.001.

16. Bleker LS, Milgrom J, Sexton-Oates A, Roseboom TJ, Gemmill AW, Holt CJ, et al. Exploring the effect of depression treatment during pregnancy on offspring's epigenetic profiles. Archives of Women's Mental Health. 2019;22(5):654-. doi: 10.1007/s00737-019-00996-y.

17. Dennis C. Training health visitors to identify and treat depressive symptoms with psychological approaches reduces postnatal depression. Evidence Based Mental Health. 2009;12(3):83-. doi: 10.1136/ebmh.12.3.83.

18. Fatori D, Matijasevitch A, Brentani H, Constantino Miguel E, Polanczyk G. 3.29 MATERNAL CARE AND ITS ASSOCIATION WITH DEPRESSION AND STRESS: A SMARTPHONE DAILY DIARY STUDY IN THE CONTEXT OF A HOME-VISITING INTERVENTION FOR ADOLESCENT MOTHERS LIVING IN ADVERSE CONDITIONS IN SÃO PAULO, BRAZIL. Journal of the American Academy of Child and Adolescent Psychiatry. 2019;58(10):S204-S. doi: 10.1016/j.jaac.2019.08.192.

19. Husain N, Lovell K, Lunat F, Atif N, Bhokari A, Bhojani I, et al. Exploratory randomized controlled trial of a group psychological intervention for postnatal depression in British mothers of South Asian origin. European Psychiatry. 2016;33:S279-S. doi: 10.1016/j.eurpsy.2016.01.746.

20. Jesse DE, Bian H, Feldhousen EB, Newton ER, Gaynes BN, Hollon SD. The role of mediators in reducing antepartum depressive symptoms in rural low-income women receiving a culturally tailored cognitive behavioral intervention. Journal of Midwifery and Women's Health. 2016;61(5):659-60. doi: 10.1111/jmwh.12561.

21. Jesse DE, Blanchard A, Bunch S, Dolbier C, Hodgson J, Swanson MS. A pilot study to reduce risk for antepartum depression among women in a public health prenatal clinic. Issues in Mental Health Nursing. 2010;31(5):355-64. PMID: 2010-08031-008. doi: 10.3109/01612840903427831.

22. Jesse DE, Gaynes BN, Feldhousen EB, Newton ER, Bunch S, Hollon SD. Performance of a Culturally Tailored Cognitive-Behavioral Intervention Integrated in a Public Health Setting to Reduce Risk of Antepartum Depression: A Randomized Controlled Trial. Journal of Midwifery and Women's Health. 2015;60(5):578-92. doi: 10.1111/jmwh.12308.

23. Junge-Hoffmeister J, Bittner A, Richter J, Schultz U, Joraschky P, Weidner K. Preventing peripartal psychosomatic symptoms in pregnant women - Is there an impact on pregnancy and birth complications or neonatal outcomes. Archives of Women's Mental Health. 2011;14:S32-S. doi: 10.1007/s00737-010-0203-1.

24. Le H-N, Perry DF, Stuart EA. Randomized controlled trial of a preventive intervention for perinatal depression in high-risk Latinas. Journal of Consulting and Clinical Psychology. 2011;79(2):135-41. doi: 10.1037/a0022492.

25. Lowndes TA, Egan SJ, McEvoy PM. Efficacy of brief guided self-help cognitive behavioral treatment for perfectionism in reducing perinatal depression and anxiety: a randomized controlled trial. Cognitive behaviour therapy. 2019;48(2):106-20. doi: 10.1080/16506073.2018.1490810.

26. Matthey S. Telephone based peer support can reduce postnatal depression in women at high risk. Evidence Based Mental Health. 2009;12(3):82-. doi: 10.1136/ebmh.12.3.82.

27. Milgrom J, Holt C, Holt CJ, Ross J, Ericksen J, Gemmill AW. Feasibility study and pilot randomised trial of an antenatal depression treatment with infant follow-up. Archives of Womens Mental Health. 2015;18(5):717-30. doi: 10.1007/s00737-015-0512-5.

28. Milgrom J, Holt C, Schembri C, Gemmill A. Development of a CBT program for depression during pregnancy-beating the blues before birth. Archives of Women's Mental Health. 2015;18(2):392-. doi: 10.1007/s00737-014-0488-6.

29. Milgrom J, Holt C, Schembri C, Gemmill A. Pilot results on child outcomes of antenatal depression treatment. Archives of Women's Mental Health. 2015;18(2):372-. doi: 10.1007/s00737-014-0488-6.

30. Milgrom J, Schembri C, Ericksen J, Ross J, Gemmill AW. Towards parenthood: An antenatal intervention to reduce depression, anxiety and parenting difficulties. Journal of Affective Disorders. 2011;130(3):385-94. PMID: 2011-09341-004. doi: 10.1016/j.jad.2010.10.045.

31. Netsi E, Jonathan E, Heather O, Alison B, Paul R. A pilot randomized controlled trial of cognitive behavioural therapy for women with antenatal depression: Infant temperament and sleep. Archives of Women's Mental Health. 2015;18(2):400-. doi: 10.1007/s00737-014-0488-6.

32. Ngai FW. Telephone-based cognitive-behavioral therapy on postnatal depression and quality of life. BJOG: An International Journal of Obstetrics and Gynaecology. 2018;125:18-. doi: 10.1111/1471-0528.15131.

33. O'Mahen H, Himle JA, Fedock G, Henshaw E, Flynn H. A pilot randomized controlled trial of cognitive behavioral therapy for perinatal depression adapted for women with low incomes. Depression & Anxiety (1091-4269). 2013;30(7):679-87. doi: 10.1002/da.22050.

34. Ramezani S, Khosravi A, Motaghi Z, Hamidzadeh A, Mousavi SA. The effect of cognitive-behavioural and solution-focused counselling on prevention of postpartum depression in nulliparous pregnant women. Journal of Reproductive and Infant Psychology. 2017;35(2):172-82. PMID: 2017-11695-007. doi: 10.1080/02646838.2016.1266470.

35. Tandon SD, Perry DF, Mendelson T, Kemp K, Leis JA. Preventing perinatal depression in low-income home visiting clients: A randomized controlled trial. Journal of Consulting and Clinical Psychology. 2011;79(5):707-12. doi: 10.1037/a0024895.

36. Timpano KR, Abramowitz JS, Mahaffey BL, Mitchell MA, Schmidt NB. Efficacy of a prevention program for postpartum obsessive-compulsive symptoms. Journal of Psychiatric Research. 2011;45(11):1511-7. doi: 10.1016/j.jpsychires.2011.06.015.

37. Van Ravesteyn LM, Kamperman AM, Schneider TAJ, Raats ME, Steegers EAP, Tiemeier H, et al. Group-based multicomponent treatment to reduce depressive symptoms in women with co-morbid psychiatric and psychosocial problems during pregnancy: A randomized controlled trial. Journal of Affective Disorders. 2018;226:36-44. doi: 10.1016/j.jad.2017.09.019.

38. Verbeek T, Bockting CLH, Meijer JL, Beijers, Van Pampus MG, Burger H. Psychological treatment of antenatal depression and anxiety: Effects on obstetric outcomes. Archives of Women's Mental Health. 2015;18(2):339-40. doi: 10.1007/s00737-014-0488-6.

39. Weems CF, Scheeringa MS. Maternal depression and treatment gains following a cognitive behavioral intervention for posttraumatic stress in preschool children. Journal of Anxiety Disorders. 2013;27(1):140-6. PMID: 2013-05917-018. doi: 10.1016/j.janxdis.2012.11.003.

40. Hayes BA, Muller R. Prenatal depression: a randomized controlled trial in the emotional health of primiparous women. Research and Theory for Nursing Practice: An International Journal. 2004;18(2-3):165-83. doi: 10.1891/rtnp.18.2.165.61277.

41. Bagnall KM. Long-term follow-up of NetmumsHWD: A feasibility randomised controlled trial of telephone supported online behavioural activation for postnatal depression at 16 months post-randomisation: University of Exeter (United Kingdom); 2014.

42. Brugha T, Smith J, Bankart J, Lovett C, Austin J, Patterson M, et al. Pregnancy and wellbeing study (PAWS). Taking action with midwives and perinatal primary care. Archives of Women's Mental Health. 2013;16:S2-S3. doi: 10.1007/s00737-013-0355-x.

43. Dennis CL. The effect of peer support on postpartum depression: A pilot randomized controlled trial. Canadian Journal of Psychiatry. 2003;48(2):115-24. doi: 10.1177/070674370304800209.

44. Dennis CL, Hodnett E, Kenton L, Weston J, Zupancic J, Stewart DE, et al. Effect of peer support on prevention of postnatal depression among high risk women: Multisite randomised controlled trial. BMJ (Online). 2009;338(7689):280-3. doi: 10.1136/bmj.a3064.

45. Fonseca A, Alves S, Monteiro F, Gorayeb R, Canavarro MC. Be a Mom, a Web-Based Intervention to Prevent Postpartum Depression: Results From a Pilot Randomized Controlled Trial. Behavior Therapy. 2020;51(4):616-33. doi: 10.1016/j.beth.2019.09.007.

46. Fonseca A, Monteiro F, Alves S, Gorayeb R, Canavarro MC. Be a mom, a web-based intervention to prevent postpartum depression: The enhancement of self-regulatory skills and its association with postpartum depressive symptoms. Frontiers in Psychology. 2019;10. doi: 10.3389/fpsyg.2019.00265.

47. Jannati N, Mazhari S, Ahmadian L, Mirzaee M. Effectiveness of an app-based cognitive behavioral therapy program for postpartum depression in primary care: A randomized controlled trial. International Journal of Medical Informatics. 2020;141. doi: 10.1016/j.ijmedinf.2020.104145.

48. King E. The effectiveness of an internet-based stress management program in the prevention of postpartum stress, anxiety and depression for new mothers: ProQuest Information & Learning; 2009.

49. Leung SS, Lee AM, Wong DF, Wong CM, Leung KY, Chiang VC, et al. A brief group intervention using a cognitive-behavioural approach to reduce postnatal depressive symptoms: a randomised controlled trial. Hong Kong medical journal = Xianggang yi xue za zhi. 2016;22:S4-S8.

50. Loughnan SA, Butler C, Sie AA, Grierson AB, Chen AZ, Hobbs MJ, et al. A randomised controlled trial of 'MUMentum postnatal': Internet-delivered cognitive behavioural therapy for anxiety and depression in postpartum women. Behaviour Research and Therapy. 2019;116:94-103. doi: 10.1016/j.brat.2019.03.001.

51. Metcalf CA. Decentering and self-compassion: A randomized controlled trial of target engagement in mindful mood balance for moms: ProQuest Information & Learning; 2020.

52. Milgrom J, Danaher BG, Gemmill AW, Holt C, Holt CJ, Seeley JR, et al. Internet Cognitive Behavioral Therapy for Women With Postnatal Depression: A Randomized Controlled Trial of MumMoodBooster. Journal of Medical Internet Research. 2016;18(3):18-. doi: 10.2196/jmir.4993.

53. Milgrom J, Danaher BG, Gemmill AW, Holt C, Holt CJ, Seeley JR, et al. Internet cognitive behavioural therapy for women with postnatal depression: A randomized controlled trial of MumMoodBooster. Archives of Women's Mental Health. 2020;23(2):293-4. doi: 10.1007/s00737-019-00953-9.

54. Netsi E, Evans J, Wulff K, O’Mahen H, Ramchandani PG, O'Mahen H, et al. Infant outcomes following treatment of antenatal depression: Findings from a pilot randomized controlled trial. Journal of Affective Disorders. 2015;188:252-6. doi: 10.1016/j.jad.2015.08.055.

55. Ngai FW, Wong PW-C, Chung KF, Leung KY. The effect of telephone-based cognitive-behavioural therapy on parenting stress: A randomised controlled trial. Journal of Psychosomatic Research. 2016;86:34-8. doi: 10.1016/j.jpsychores.2016.03.016.

56. O'Mahen HA, Richards DA, Woodford J, Wilkinson E, McGinley J, Taylor RS, et al. Netmums: a phase II randomized controlled trial of a guided Internet behavioural activation treatment for postpartum depression. Psychological medicine. 2014;44(8):1675-89. doi: 10.1017/s0033291713002092.

57. O'Mahen HA, Woodford J, McGinley J, Warren FC, Richards DA, Lynch TR, et al. Internet-based behavioral activation—Treatment for postnatal depression (Netmums): A randomized controlled trial. Journal of Affective Disorders. 2013;150(3):814-22. doi: 10.1016/j.jad.2013.03.005.

58. Pugh NE, Hadjistavropoulos HD, Dirkse D. A Randomised Controlled Trial of Therapist-Assisted, Internet-Delivered Cognitive Behavior Therapy for Women with Maternal Depression. PLoS ONE. 2016;11(3):13-. doi: 10.1371/journal.pone.0149186.

59. Seeley JR, Sheeber LB, Feil EG, Leve C, Davis B, Sorensen E, et al. Mediation analyses of Internet-facilitated cognitive behavioral intervention for maternal depression. Cognitive behaviour therapy. 2019;48(4):337-52. doi: 10.1080/16506073.2018.1513554.

60. Sembi S. Mums4Mums : structured telephone peer-support for women experiencing postnatal depression : a pilot RCT to test its clinical effectiveness [Electronic Thesis or Dissertation]2018.

61. Tandon SD, Leis JA, Mendelson T, Perry DF, Kemp K. Six-Month Outcomes from a Randomized Controlled Trial to Prevent Perinatal Depression in Low-Income Home Visiting Clients. Maternal and child health journal. 2014;18(4):873-81. doi: 10.1007/s10995-013-1313-y.

62. Thitipitchayanant K, Somrongthong R, Kumar R, Kanchanakharn N. Effectiveness of self-empowerment-affirmation-relaxation (Self-EAR) program for postpartum blues mothers: A randomize controlled trial. Pakistan Journal of Medical Sciences. 2018;34(6):1488-93. doi: 10.12669/pjms.346.15986.

63. Van Lieshout RJ, Layton H, Savoy CD, Brown JSL, Ferro MA, Streiner DL, et al. Effect of Online 1-Day Cognitive Behavioral Therapy-Based Workshops plus Usual Care vs Usual Care Alone for Postpartum Depression: A Randomized Clinical Trial. JAMA Psychiatry. 2021. doi: 10.1001/jamapsychiatry.2021.2488.

64. Vigod SN, Slyfield Cook G, Macdonald K, Hussain-Shamsy N, Brown HK, de Oliveira C, et al. Mother matters: Pilot randomized wait‐list controlled trial of an online therapist‐facilitated discussion board and support group for postpartum depression symptoms. Depression and Anxiety. 2021;38(8):816-25. PMID: 2021-43970-001. doi: 10.1002/da.23163.

65. Wozney L, Olthuis J, Lingley-Pottie P, McGrath PJ, Chaplin W, Elgar F, et al. Strongest Families™ Managing Our Mood (MOM): a randomized controlled trial of a distance intervention for women with postpartum depression. Archives of Women's Mental Health. 2017;20(4):525-37. doi: 10.1007/s00737-017-0732-y.

66. Baylis R, Ekdahl J, Haines H, Rubertsson C. Women's experiences of internet-delivered Cognitive Behaviour Therapy (iCBT) for Fear of Birth. Women and Birth. 2020;33(3):E227-E33. doi: 10.1016/j.wombi.2019.05.006.

67. Bei B, Neemia D, Shen L, Fulgoni C, Blumfield ML, Drummond SP, et al. A brief, automated cognitive behavioral program prevents sleep disturbance and insomnia in late pregnancy: A randomized controlled trial. Sleep. 2018;41:A151-A.

68. Cain MA, Brumley J, Beauchamp W, Louis-Jacques A, Drerup M, Louis JM. A pilot study of cognitive behavioral therapy for insomnia delivered in pregnancy. Sleep. 2016;39:A214-A.

69. Doty MS, Chen HY, Ajishegiri O, Sibai BM, Blackwell SC, Chauhan SP. 251 Mindful meditation for anxiety in individuals admitted to the antepartum unit: a randomized controlled trial. American Journal of Obstetrics and Gynecology. 2021;224(2):S166. doi: 10.1016/j.ajog.2020.12.273.

70. Fenwick J, Toohill J, Gamble J, Creedy DK, Buist A, Turkstra E, et al. Effects of a midwife psycho-education intervention to reduce childbirth fear on women's birth outcomes and postpartum psychological wellbeing. BMC Pregnancy and Childbirth. 2015;15:8-. doi: 10.1186/s12884-015-0721-y.

71. Huang L, Shen Q, Fang Q, Zheng X. Effects of Internet-Based Support Program on Parenting Outcomes for Primiparous Women: A Pilot Study. International journal of environmental research and public health. 2021;18(9). PMID: 33919112. doi: 10.3390/ijerph18094402.

72. Kalmbach DA, Cheng PL, O'Brien LM, Swanson LM, Sangha R, Sen S, et al. A randomized controlled trial of digital cognitive behavioral therapy for insomnia in pregnant women. Sleep Medicine. 2020;72:82-92. doi: 10.1016/j.sleep.2020.03.016.

73. Krusche A, Dymond M, Murphy SE, Crane C. Mindfulness for pregnancy: A randomised controlled study of online mindfulness during pregnancy. Midwifery. 2018;65:51-7. doi: 10.1016/j.midw.2018.07.005.

74. Levine MD, Marcus MD, Kalarchian MA, Cheng Y. Strategies to Avoid Returning to Smoking (STARTS): A randomized controlled trial of postpartum smoking relapse prevention interventions. Contemporary Clinical Trials. 2013;36(2):565-73. doi: 10.1016/j.cct.2013.10.002.

75. Manber R, Bei B, Simpson N, Rangel E. Cognitive behavioral therapy for perinatal insomnia: Effects on postpartum depressive symptoms. Sleep. 2020;43(SUPPL 1):A204-A. doi: 10.1093/sleep/zsaa056.531 LK - <http://sfx.nelliportaali.fi/nelli30b?sid=EMBASE&issn=15509109&id=doi:10.1093%2Fsleep%2Fzsaa056.531&atitle=Cognitive+behavioral+therapy+for+perinatal+insomnia%3A+Effects+on+postpartum+depressive+symptoms&stitle=Sleep&title=Sleep&volume=43&issue=SUPPL+1&spage=A204&epage=&aulast=Manber&aufirst=R.&auinit=R.&aufull=Manber+R.&coden=&isbn=&pages=A204-&date=2020&auinit1=R&auinitm>=.

76. Rangel E, Asarnow L, Simpson N, Manber R. Predictors of session attendance in a rct for CBT-I for perinatal insomnia. Sleep. 2020;43(SUPPL 1):A191-A. doi: 10.1093/sleep/zsaa056.496 LK - <http://sfx.nelliportaali.fi/nelli30b?sid=EMBASE&issn=15509109&id=doi:10.1093%2Fsleep%2Fzsaa056.496&atitle=Predictors+of+session+attendance+in+a+rct+for+CBT-I+for+perinatal+insomnia&stitle=Sleep&title=Sleep&volume=43&issue=SUPPL+1&spage=A191&epage=&aulast=Rangel&aufirst=E.&auinit=E.&aufull=Rangel+E.&coden=&isbn=&pages=A191-&date=2020&auinit1=E&auinitm>=.

77. Toohill J, Fenwick J, Gamble J, Creedy DK, Buist A, Turkstra E, et al. A randomized controlled trial of a psycho-education intervention by midwives in reducing childbirth fear in pregnant women. Birth (Berkeley, Calif). 2014;41(4):384-94. doi: 10.1111/birt.12136.

78. Van Ravesteyn L, Van Ravesteyn LM, Birnie E, Schneider AJ, Lambregtse-Van Den Berg MP. Group-based multicomponent psychotherapy for pregnant women with a mental disorder. Archives of Women's Mental Health. 2015;18(2):391-. doi: 10.1007/s00737-014-0488-6.

79. Baker-Ericzén MJ, Connelly CD, Hazen AL, Dueñas C, Landsverk JA, Horwitz SM. A collaborative care telemedicine intervention to overcome treatment barriers for Latina women with depression during the perinatal period. Families, Systems, & Health. 2012;30(3):224-40. PMID: 2012-16098-001. doi: 10.1037/a0028750.

80. Biggs L, Forster D, Shafiei T, McLachlan H, Smith T, Wyett C, et al. IVY: InVestigating an online community of support for emotional health in pregnancy. Women and Birth. 2018;31:S37-S. doi: 10.1016/j.wombi.2018.08.112.

81. Bleker LS, Milgrom J, Parker D, Gemmill AW, Holt CJ, Connelly A, et al. Brain Magnetic Resonance Imaging Findings in Children after Antenatal Maternal Depression Treatment, a Longitudinal Study Built on a Pilot Randomized Controlled Trial. International Journal of Environmental Research and Public Health. 2019;16(10). doi: 10.3390/ijerph16101816.

82. Bleker LS, Milgrom J, Sexton-Oates A, Parker D, Roseboom TJ, Gemmill AW, et al. Cognitive Behavioral Therapy for Antenatal Depression in a Pilot Randomized Controlled Trial and Effects on Neurobiological, Behavioral and Cognitive Outcomes in Offspring 3–7 Years Postpartum: A Perspective Article on Study Findings, Limitations and Futu. Frontiers in Psychiatry. 2020;11. doi: 10.3389/fpsyt.2020.00034.

83. Butler C, Loughnan S, Newby J, Andrews G. Assessing and managing risk online: A clinician's perspective on internet-delivered intervention, 'MUMentum', for perinatal distress, anxiety, and depression. Archives of Women's Mental Health. 2019;22(5):699-. doi: 10.1007/s00737-019-00996-y.

84. Clifton J, Parent J, Worrall G, Seehuus M, Evans M, Forehand R, et al. An internet-based mind/body intervention to mitigate distress in women experiencing infertility: A randomized pilot trial. Fertility and Sterility. 2016;106:e62-e.

85. Drozd F, Haga SM, Brendryen H, Slinning K. An Internet-Based Intervention (Mamma Mia) for Postpartum Depression: Mapping the Development from Theory to Practice. Jmir Research Protocols. 2015;4(4):17-. doi: 10.2196/resprot.4858.

86. Haga SM, Drozd F, Brendryen H, Slinning K. Mamma Mia: A feasibility study of a web-based intervention to reduce the risk of postpartum depression and enhance subjective well-being. Journal of Medical Internet Research. 2013;15(8). doi: 10.2196/resprot.2659.

87. Hulsbosch LP, Nyklíček I, Potharst ES, Meems M, Boekhorst MGBM, Pop VJM. Online mindfulness-based intervention for women with pregnancy distress: Design of a randomized controlled trial. BMC Pregnancy and Childbirth. 2020;20(1). doi: 10.1186/s12884-020-2843-0.

88. Kammerer M, Castro RTA, Glover V, Ramchandani P, Bennett P. A randomized controlled trial of internet based cognitive behavioural therapy (CBT) versus treatment as usual for pregnant women with high levels of depression at queen charlotte's hospital. Archives of Women's Mental Health. 2013;16:S18-S. doi: 10.1007/s00737-013-0355-x.

89. Kantrowitz-Gordon I, McCurry SM, Landis C, Lee R, Wi D. Online prenatal trial in mindfulness sleep management (OPTIMISM): a pilot randomized controlled trial for insomnia in pregnancy. Sleep Medicine. 2019;64:S188-S9. doi: 10.1016/j.sleep.2019.11.522.

90. Kim DR, Hantsoo L, Thase ME, Sammel M, Epperson CN. Computer-Assisted Cognitive Behavioral Therapy for Pregnant Women with Major Depressive Disorder. Journal of Womens Health. 2014;23(10):842-8. doi: 10.1089/jwh.2014.4867.

91. Lovell K, Chaudhry N, Masood Y, Lunat F, Atif N, Syed A, et al. Exploratory rct of a group psychological intervention for postnatal depression in british mother's of south asian origin-post intervention qualitative study. Archives of Women's Mental Health. 2015;18(2):336-7. doi: 10.1007/s00737-014-0488-6.

92. McCarter DE, Demidenko E, Hegel MT. Measuring outcomes of digital technology-assisted nursing postpartum: A randomized controlled trial. Journal of advanced nursing. 2018;74(9):2207-17. doi: 10.1111/jan.13716.

93. Milgrom J, Danaher B, Schembri C, Seeley J, Ericksen J, Gemmill A, et al. Web-based cognitive behavioural therapy for postnatal depression. Archives of Women's Mental Health. 2013;16:S48-S. doi: 10.1007/s00737-013-0355-x.

94. Milgrom J, Danaher B, Seeley J, Schembri C, Health A, Ericksen J, et al. Mummoodbooster-an interactive internet treatment for postnatal depression. Archives of Women's Mental Health. 2015;18(2):334-5. doi: 10.1007/s00737-014-0488-6.

95. Milgrom J, Gemmill A, Holt C, Holt C, Oliva J. Perinatal e-mental health support: Evidence and challenges in translation to practice. Archives of Women's Mental Health. 2019;22(5):681-2. doi: 10.1007/s00737-019-00996-y.

96. Morrell CJ, Ricketts T, Tudor K, Williams C, Curran J, Barkham M. Training health visitors in cognitive behavioural and person-centred approaches for depression in postnatal women as part of a cluster randomised trial and economic evaluation in primary care: the PoNDER trial. Primary health care research & development. 2011;12(1):11-20. doi: 10.1017/s1463423610000344.

97. Simon GE. CBT improves maternal perinatal depression in rural Pakistan. Evidence Based Mental Health. 2009;12(2):45-. PMID: 105528641. Language: English. Entry Date: 20090529. Revision Date: 20200708. Publication Type: Journal Article. doi: 10.1136/ebmh.12.2.45.

98. Trevillion K, Hunter MS, Bick D, Byford S, Pickles A, Milgrom J, et al. Depression: An exploratory parallel-group randomised controlled trial of antenatal guided self help for women (dawn trial). Archives of Women's Mental Health. 2015;18(2):356-. doi: 10.1007/s00737-014-0488-6.

99. Wajid A, van Zanten SV, Mughal MK, Biringer A, Austin M-P, Vermeyden L, et al. Adversity in childhood and depression in pregnancy. Archives of Women's Mental Health. 2020;23(2):169-80. doi: 10.1007/s00737-019-00966-4.

100. Bullock LF, Wells JE, Duff GB, Hornblow AR. Telephone support for pregnant women: outcome in late pregnancy. The New Zealand medical journal. 1995;108(1012):476-8.

101. Fancourt D, Perkins R. COMBINED GROUP AND TECHNOLOGY-SUPPORTED SINGING INTERVENTIONS FOR POSTNATAL DEPRESSION: PSYCHOLOGICAL AND BIOLOGICAL RESULTS FROM A THREE-ARM RANDOMISED CONTROL TRIAL. Psychosomatic Medicine. 2017;79(4):A142-A3.

102. Giallo R, Cooklin A, Dunning M, Seymour M. The efficacy of an intervention for the management of postpartum fatigue. Journal of Obstetric, Gynecologic, & Neonatal Nursing: Clinical Scholarship for the Care of Women, Childbearing Families, & Newborns. 2014;43(5):598-613. doi: 10.1111/1552-6909.12489.

103. Loughnan S. Regaining MUMentum: Findings from two randomized controlled trials evaluating brief internet cognitive behavioral therapy for perinatal distress, anxiety, and depression. Archives of Women's Mental Health. 2019;22(5):682-. doi: 10.1007/s00737-019-00996-y.

104. Loughnan SAM, Newby J, Andrews G, Butler C. Regaining 'MUMentum': Randomized controlled trial of online CBT for perinatal distress, anxiety, and depression. Archives of Women's Mental Health. 2019;22(5):704-. doi: 10.1007/s00737-019-00996-y.
