# Supplementary material for: Digitalized Cognitive Behavioral Interventions for Depressive Symptoms During Pregnancy: Systematic Review
Source: J Med Internet Res. 2022 Feb 23;24(2):e33337. doi: 10.2196/33337 (PMC8908191; doi:10.2196/33337)
Supplement: Multimedia Appendix 3 [file jmir_v24i2e33337_app3.docx]

**Supplementary File 3: Attempts to contact authors**

We contacted the authors for additional information of the study. Reminder emails were sent a week after the first email.

| **Study** | **Contacted** | **Replied** | **Provided additional information** | **Notes** |
| --- | --- | --- | --- | --- |
| Barrera et al. (2015) | Yes | Yes | Yes |  |
| Guo et al. (2020) | Yes | No | No | Only corresponding authors’ email and affiliation available. Not possible to retrieve a valid email address of other authors |
